# Ab Initio Crystal Structure Prediction of the Energetic Materials LLM-105, RDX, and HMX

**DOI:** 10.1021/acs.cgd.3c00027

**Published:** 2023-08-17

**Authors:** Dana O’Connor, Imanuel Bier, Rithwik Tom, Anna M. Hiszpanski, Brad A. Steele, Noa Marom

**Affiliations:** † Department of Materials Science and Engineering, Carnegie Mellon University, Pittsburgh, Pennsylvania 15213, United States; ‡ Department of Physics, Carnegie Mellon University, Pittsburgh, Pennsylvania 15213, United States; § Materials Science Division, Lawrence Livermore National Laboratory, Livermore, California 94550, United States; ∥ Department of Chemistry, Carnegie Mellon University, Pittsburgh, Pennsylvania 15213, United States

## Abstract

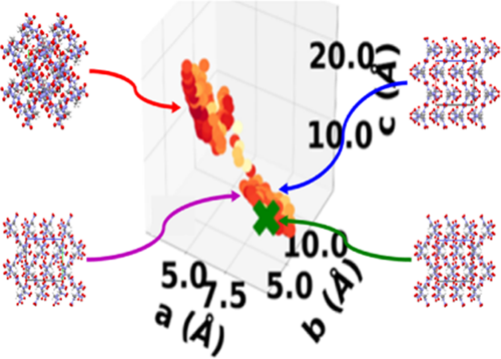

Crystal structure prediction (CSP) is performed for the energetic materials (EMs) LLM-105 and α-RDX, as well as the α and β conformational polymorphs of 1,3,5,7-tetranitro-1,3,5,7-tetraazacyclooctane (HMX), using the genetic algorithm (GA) code, GAtor, and its associated random structure generator, Genarris. Genarris and GAtor successfully generate the experimental structures of all targets. GAtor’s symmetric crossover scheme, where the space group symmetries of parent structures are treated as genes inherited by offspring, is found to be particularly effective. However, conducting several GA runs with different settings is still important for achieving diverse samplings of the potential energy surface. For LLM-105 and α-RDX, the experimental structure is ranked as the most stable, with all of the dispersion-inclusive density functional theory (DFT) methods used here. For HMX, the α form was persistently ranked as more stable than the β form, in contrast to experimental observations, even when correcting for vibrational contributions and thermal expansion. This may be attributed to insufficient accuracy of dispersion-inclusive DFT methods or to kinetic effects not considered here. In general, the ranking of some putative structures is found to be sensitive to the choice of the DFT functional and the dispersion method. For LLM-105, GAtor generates a putative structure with a layered packing motif, which is desirable thanks to its correlation with low sensitivity. Our results demonstrate that CSP is a useful tool for studying the ubiquitous polymorphism of EMs and shows promise of becoming an integral part of the EM development pipeline.

## Abstract 2

Crystal structure prediction (CSP) is performed for the energetic materials LLM-105 and α-RDX as well as the α and β conformational polymorphs of HMX using the genetic algorithm GAtor and its associated random structure generator Genarris.

## Introduction

Molecular crystals are a broad class of materials that consist of molecules bound together through intermolecular interactions in a periodic lattice. Molecular crystals have many practical applications including organic electronics,^1,2^ pharmaceuticals,^3,4^ and energetic materials (EMs).^5,6^ The weak intermolecular interactions allows crystals of the same molecule to pack in multiple different structures, known as polymorphs. Molecular crystals often exhibit polymorphism,^7,8^ and different polymorphs can have profoundly different properties.

For energetic materials (EMs), polymorphism is ubiquitous.^9−11^ For example, 1,3,5,7-tetranitro-1,3,5,7-tetraazacyclooctane (HMX) has four known polymorphs. The β form is the most stable at ambient conditions, whereas α and δ are high-temperature polymorphs. The final polymorph, γ, is a hemihydrate.^12−14^ The shock sensitivity of the four HMX polymorphs follows the order δ > γ > α > β.^12^ The polymorphism of HMX is also relevant for understanding the kinetics for the time to explosion.^15,16^ Polymorphism is therefore important for understanding the properties of EMs.

For the shock-induced ignition of EMs, the material is rapidly strained under GPa-range pressures, which can also induce significant heating in the material. In order to characterize how EMs behave under such extreme conditions, diamond anvil cell (DAC) experiments are often performed on EMs concomitant with X-ray diffraction (XRD) or vibrational spectroscopic measurements. The data is used to parameterize an equation of state (EOS) that is used to model shock-induced ignition.^17^ Therefore, there have been extensive efforts to determine the high-pressure unreacted EOS of EMs, many of which have shown evidence of pressure-induced polymorphic phase transitions. High-pressure polymorphic phase transitions have been studied both experimentally and theoretically for 1,3,5-triamino-2,4,6-trinitrobenzene (TATB),^18−20^ ammonium nitrate,^21,22^ dihydroxylammonium 5,5′-bistetrazole-1,1’-diolate (TKX-50),^23,24^ 1,3,5-trinitroperhydro-1,3,5-triazine (RDX),^25−29^ HMX,^14,30−35^ hexanitrohexaazaisowurtzitane (CL-20),^36−39^ 2,6-diamino-3,5-dinitropyrazine-1-oxide (LLM-105),^40−44^ pentaerythritol tetranitrate (PETN),^45,46^ and 1,1-diamino-2,2-dinitroethylene (FOX-7),^47^ to list a few. Some polymorphs have been reported without crystallographic information such as δ-RDX (18 GPa),^26^ ζ-RDX (27.6 GPa),^48^ δ-FOX-7 (210 °C),^49^ PETN-III (8.5 GPa),^50^ and PETN-IV (136 °C, 9.2 GPa).^50^ In some cases, there has been debate over the occurrence of a phase transition. For example, crystals of β-HMX have been probed under quasi-hydrostatic conditions up to 45 GPa using powder XRD and micro-Raman spectroscopy.^30^ The results suggested a phase transition at 12 GPa with no abrupt volume change, later attributed to the epsilon phase,^31^ and a discontinuous volume change of 4% at 27 GPa.^30^ However, more recent studies reported conflicting results regarding the existence of a phase transition above 27 GPa.^32−35^ For LLM-105, one first-principles investigation^41^ indicated a series of structural phase transitions at 8, 17, 25, and 42 GPa based on irregular changes of lattice parameters, while another indicated just one phase transition at 30 GPa.^44^ Manaa et al.^42^ performed first-principles molecular dynamics simulations and concluded that the ambient pressure phase of LLM-105 remains stable up to 45 GPa. Experiments by Stavrou et al. found that the ambient phase remains stable up to 20 GPa,^43^ whereas Xu et al. observed a structural phase transition at about 30 GPa, based on the pressure-dependent Raman and infrared spectra.^51^

Understanding the high-pressure polymorphism of EMs could be greatly assisted by performing computational crystal structure prediction (CSP) to identify potential polymorphs. CSP has been used extensively to study polymorphism in various molecular crystals, e.g., in refs (19, 22, 52−56). CSP simulations attempt to find the global minimum of the total energy energy as a function of the positions of the atoms and lattice parameters. The simulations are almost always performed at 0 K, but the same principle applies at nonzero temperatures, as well as at elevated pressure.^57−59^ One challenge is that the search space is high-dimensional. The potential energy surface (PES) is a multidimensional hypersurface that describes the energy of all possible atomic positions and lattice parameters. The PES contains many local minima, whose number scales exponentially with system size. The exponential scaling was derived by Stillinger^60^ and demonstrated for argon clusters using a Lennard-Jones potential.^61^ For molecular crystals, the independent degrees of freedom include the lattice parameters and angles of the unit cell, the number of molecules per unit cell, the positions and orientations of the molecules in the cell, and, for molecules with flexible bonds, all independent torsion angles. This amounts to a high-dimensional configuration space.

Pickard and Needs have claimed that CSP can be performed using just a random sampling methodology.^62^ One reason is that regions of the PES where the atoms are too close together, or too far apart, are irrelevant because they contain unstable structures. Another reason is that the PES can be divided into basins of attraction, which are sets of points where downhill relaxation leads to the same energy minima. It has been posited that basins of attraction with lower-energy minima tend to have larger hypervolumes on the PES and thus a higher probability of being found. However, this has not always been found to be the case.^63−65^ For example, the experimental crystal structure of 1,3-diamino-2,4,6-trinitrobenzene (DATB) has been found to have a relatively narrow basin compared to TATB.^55^ The size of the regions of the PES to be searched for molecular CSP can be reduced by applying physical assumptions. The first is an estimate of the solid-form volume of a molecule, which can be obtained, e.g., by a machine learned model.^66^ This can be used to define the range of unit cell volumes to be searched. The second assumption is that typical intermolecular close contacts depend on the nature of and strength of intermolecular interactions. Weak van der Waals (vdW) interactions are characterized by intermolecular close-contact distances close to the sum of the vdW radii of the participating atoms.^67^ Strong hydrogen-bonding interactions take place at significantly shorter distances.^68^ Restricting the intermolecular distances to be searched significantly reduces the number of molecular packing arrangements that need to be considered. However, even with these simplifying assumptions, the size of the PES to be searched is still immense.

The search can be accelerated considerably by applying smart sampling strategies rather than random generation. For example, a genetic algorithm (GA)^52,63,69−71^ uses information about low-energy structures in order to propagate structural features associated with relatively stable configurations of molecules to produce progressively improved structures until the global minimum is found. GAs are inspired by the evolutionary principle of survival of the fittest, whereby structures that have a higher fitness are assigned a higher likelihood to be chosen to create offspring.^52,63,72^ The GA starts from an initial population of structures. Parent structures are selected for mating according to a probability distribution based on their fitness. Child structures are generated through crossover and mutation operations, which blend or modify the molecular positions and orientations, and the lattice parameters of parent structures. Mutation operations alter a single parent structure, whereas crossover operations combine the properties of two parent structures. After generation, the fitness of the child structure is evaluated and it is added to the pool of available structures for mating (unless it is a duplicate of an existing structure). The cycle of fitness evaluation, parent selection, and offspring generation repeats until no new lower-energy structures are found in a large number of cycles. The success of a genetic algorithm may depend on the quality of its initial population, the quality of the mutation and crossover operations, and the ability to effectively sample the full diversity of structures on the PES.^63,73^ The success of CSP is also dependent on the shape of the PES, which is system-dependent. If the global minimum is located in a narrow basin of attraction, it may be more difficult to find.^52,55,64,65,74^ Therefore, tests should be performed to evaluate the ability of CSP algorithms to effectively find the global minimum for a variety of molecular crystals.

A series of CSP blind tests have been conducted over the last two decades to test the ability of state-of-the-art methods to effectively predict the structure of molecular crystals, starting from small, rigid molecules and progressing to molecules with multiple conformational degrees of freedom, as well as salts, hydrates, and cocrystals.^75−80^ The blind tests have highlighted several successes for CSP, but also several challenges. One of the challenges is that many structures are found within a few kJ/mol of the global minimum. This generally requires a high fidelity calculation of the energy to rank the polymorphs correctly. Periodic dispersion-inclusive density functional theory (DFT) is widely used for this purpose and has become a community-accepted best practice^80^ (an alternative approach is using fragment-based quantum chemistry methods^81−84^). In some cases, the global minimum structure predicted by DFT is not the experimentally observed structure. This may be because of the limited accuracy of DFT, finite temperature effects, or synthesis conditions and kinetics.^85^ EMs are similar to the organic molecular crystals investigated in the blind tests in some respects. However, EMs may potentially represent a new challenge for CSP because they are more densely packed than typical organic molecular crystals. Moreover, most EMs comprise multiple functional groups (such as nitro and amino groups), which are not as often observed in other classes of molecules, and lead to unique intermolecular interactions.^86−89^ EMs may have large unit cells with more than 100 atoms and 8 or more molecules. The large size may be problematic due to the exponential scaling of the number of local minima in the configuration space and the increased computational cost of accurately evaluating lattice energies.

In some cases, CSP has been helpful in elucidating the high-pressure crystal structure of EMs. For TATB, a combination of high-pressure single-crystal XRD and CSP simulations revealed the crystal structure of a high-pressure monoclinic phase above 4 GPa.^19^ However, powder XRD studies have not indicated a phase transition up to 66 GPa.^18^ Furthermore, the ambient triclinic phase shows disagreement in the theoretical lattice parameters above 4 GPa.^90^ A high-pressure distortion of ammonium nitrate was also discovered using CSP simulations, and the simulated Raman spectrum of the predicted structure was shown to be in agreement with the experimental spectrum.^21,22^ Despite these efforts, the crystal structure of the high-pressure phase has not been confirmed. Aside from a handful of cases that show agreement with experiments, challenges still remain for successfully and consistently predicting the crystal structure of EMs, especially at high pressures where there are experimental difficulties in determining the structure. Therefore, CSP methods should first be tested where the crystal structure is known. After establishing the parameters and overall effectiveness of predicting known crystal structures, CSP simulations can be performed at higher pressures, where the structure may be yet unknown, with a higher degree of confidence. In addition, CSP may be used to predict potentially synthesizable polymorphs with desirable properties, such as a layered packing motif associated with decreased sensitivity.^55^

**Figure 1:**
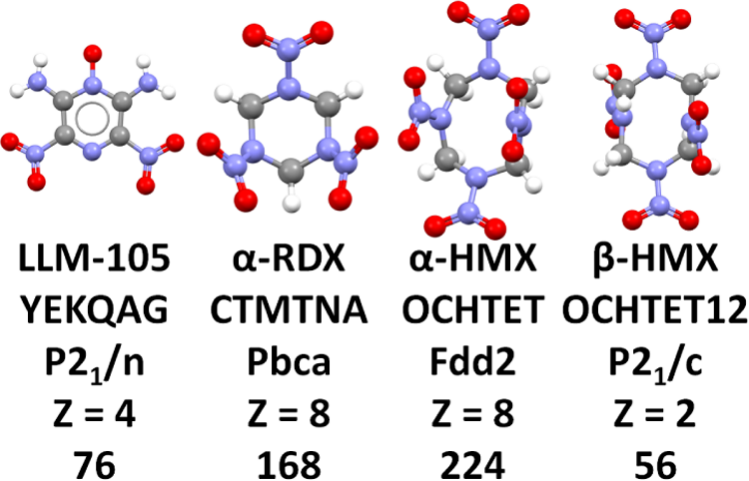
CSP targets, from left to right: 2,6-diamino-3,5-dinitropyrazine-1-oxide (LLM-105), 1,3,5-trinitroperhydro-1,3,5-triazine (RDX), and the conformers of 1,3,5,7-tetranitro-1,3,5,7-tetrazocane (HMX) found in the α- and β-forms. The corresponding CSD reference codes, space groups, number of molecules per unit cell (*Z*), and number of atoms per unit cell are listed under each molecule.

In this study, we test the ability of CSP simulations to predict the crystal structure of the EMs LLM-105, α-RDX, α-HMX, and β-HMX, shown in Figure 1. These targets represent the crystal structures of typical, widely used EMs, with the exception of LLM-105, which is a relatively new EM.^91^ To perform CSP, we use the GAtor genetic algorithm^52,63^ and its associated random structure generator, Genarris.^67,68^ All four experimental structures are generated successfully. The symmetric crossover scheme, implemented in GAtor, where space group symmetries serve as genes, proves the most successful at generating the experimental structures of all four crystals. For LLM-105 and α-RDX, the experimental structures are the most dense and are consistently ranked as the most stable with all dispersion-inclusive DFT methods used here. For HMX, although the β-form is correctly found to be the most dense, the α-form is persistently ranked as more stable, contrary to experimental observations, even when vibrational contributions and thermal expansion are taken into account. The ranking of some putative structures changes significantly, depending on the method used. For LLM-105, a putative structure is found with a layered packing motif, associated with low sensitivity. Our results demonstrate the prospects of CSP in energetic materials research.

## Methods

### Workflow Overview

The CSP workflow begins with generating an initial population using the random structure generator, Genarris.^67,68^ The initial population is fed into the genetic algorithm, GAtor.^52,63^ Several GAtor runs with different settings are performed for each target starting from the same initial pool. Then, all of the generated structures are collected and duplicates are removed. Generated structures undergo unit cell standardization and Niggly reduction.^52^ Duplicates are identified using the structure matcher functionality in pymatgen with a fractional length tolerance of 0.2, site tolerance of 0.1, and an angle tolerance of 3. The remaining unique structures within 10 kJ/mol of the global minimum are rerelaxed and reranked using increasingly accurate DFT methods.

We note that currently Genarris and GAtor work for semirigid molecules and do not explicitly account for conformational flexibility. The molecular conformation may change only to the extent possible by structural relaxation to the nearest local minimum. Hence, although some of the targets studied here have conformational polymorphs, in each search, we only generate structures with one conformer and only expect to find polymorphs with that conformer. The conformation of the monomer for each target is extracted from the experimental structure available in the Cambridge Structural Database. To account for conformational polymorphism, Genarris and GAtor runs may be seeded with different conformers, as we demonstrate for the α and β polymorphs of HMX. Structures generated with different conformers may be combined for postprocessing and final ranking.

It has been shown that the choice of the DFT functional and dispersion method can significantly affect the stability ranking of putative crystal structures.^92−99^ Therefore, postprocessing is a crucial step of the CSP workflow. Putative crystal structures are initially ranked according to their relative DFT energy per molecule, obtained with different exchange-correlation functionals and dispersion methods. Here, relaxation and reranking are performed with the Perdew, Burke, and Ernzerhof (PBE)^100^ generalized gradient approximation paired with the following dispersion methods: (i) The TkatchenkoScheffler (TS) dispersion method adds the leading order dispersion term to the DFT energy in a pairwise manner.^101^ The parameters of the correction, namely, the *C*_6_ coefficients and effective vdW radii, are calculated from first principles based on the DFT charge density. (ii) The exchange-dipole moment (XDM)^98,99,102,103^ method is a pairwise dispersion method, whose parameters are also derived from first-principles considerations, but in contrast to TS, it includes higher-order *C*_8_ and *C*_10_ terms. (iii) The many-body dispersion (MBD) method^104,105^ goes beyond the pairwise approaches. It accounts for the effect of long-range electrostatic screening on the atomic polarizabilities and for the nonpairwise-additive contributions of many-body dispersion interactions to all orders. Final ranking is performed using the PBE-based hybrid functional (PBE0)^106^ paired with the MBD dispersion method. PBE0+MBD has been shown to provide sufficient accuracy for polymorph ranking.^93−95^ In a recent benchmark, PBE+TS, PBE+XDM, PBE+MBD, and PBE0+MBD yielded mean absolute errors of 3.14, 1.04, 0.94, and 0.84 kcal/mol for lattice energies of the X23 set of molecular crystals.^99^ PBE+TS, PBE+MBD, and PBE0+MBD have been found to yield larger errors for a benchmark set of EMs than for the X23 set (the XDM method has not been benchmarked for EMs).^89^ However, to date, no alternative method has been shown to deliver better results.

Although the lattice energy is the dominant contribution to the stability of molecular crystals, it has been shown that accounting for vibrational and thermal contributions may be necessary to obtain the correct polymorph ranking.^85,95,97,107^ For the X23 set, the average vibrational and thermal contributions have been found to be 5.2 kJ/mol and 1.6 kJ/mol, respectively. For a benchmark set of 31 EMs, both the average vibrational and thermal contributions have been found to be 5.5 kJ/mol.^89^ Explicit treatment of thermal expansion has been found to have a relatively small contribution.^89^ Here, the vibrational free energy at the crystallization temperature, *T*, is added to the final PBE0+MBD energy. Treatment of thermal expansion within the quasi-harmonic approximation was only performed for the α and β polymorphs of HMX.

### Genarris

Genarris generates random structures with a distribution around a given volume in all space groups compatible with the requested number of molecules per unit cell and the molecular point group symmetry, including space groups with molecules occupying special Wyckoff positions.^68^ The unit cell volume is estimated using the PyMoVE machine learned model.^66^ Once a crystal geometry is generated, a structure check procedure detects if any distances between atoms of different molecules are too close to be physically reasonable. Structure generation continues until a user-defined number of structures is reached. The resulting structures populate the “raw” pool. Downselection from the raw pool was performed using the “robust” workflow of Genarris, which comprises two steps of clustering and selection. Clustering the population by structural similarity is performed using the affinity propagation (AP)^108^ machine learning algorithm with a radial symmetry function (RSF)^109^ representation. In the first selection step, the exemplar of each cluster is selected, based on considerations of structural diversity. For the remaining structures, single point energy evaluation is performed with DFT. Then, AP clustering is performed again and the lowest energy structure in each cluster is selected, based on stability and diversity considerations.

To decide whether two molecules are too close, the specific radius, *s*_*r*_, is used as a measure of the distance between atoms of different molecules. The *s*_*r*_ is a fraction of the sum of the van der Waals radii *r*_*A*_ and *r*_*B*_ of two atoms, *A* and *B*, belonging to different molecules. The distance, *d*_*A*,*B*_, must be such that
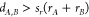
1Otherwise, the structure is rejected. In Genarris, *s*_*r*_ is a user-defined parameter with a default of 0.85. Smaller default *s*_*r*_ values have been assigned to strong hydrogen bonds, which exhibit significantly shortened intermolecular distances than typical van der Waals interactions.^68^

**Figure 2:**
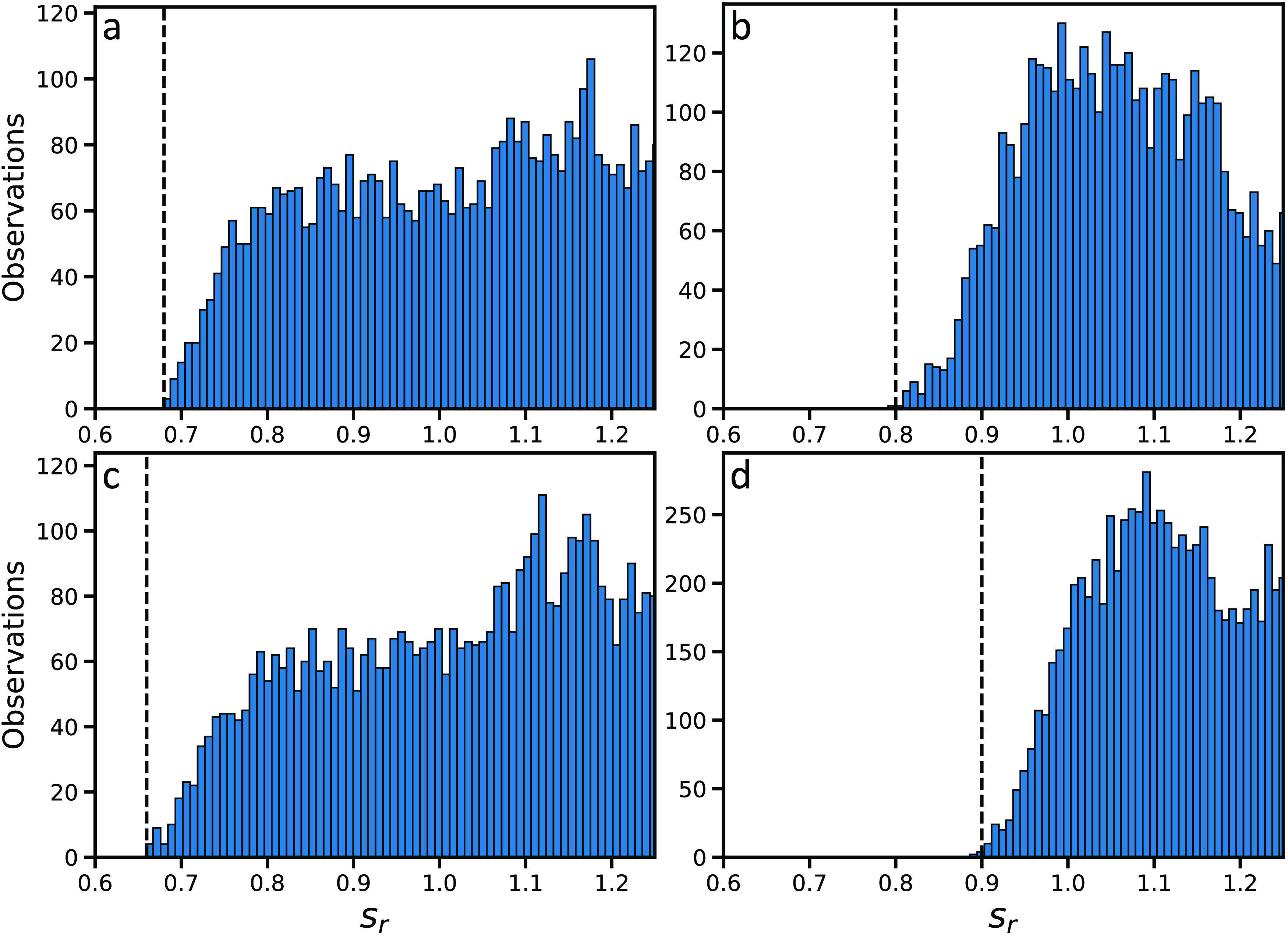
Histograms of observations of intermolecular interaction distances in structures extracted from the CSD, as a function of *s*_*r*_ for (a) the oxygen of a nitro group interacting with the hydrogen of an amine group, (b) the oxygen of a nitro group interacting with a hydrogen bonded to carbon, (c) the oxygen of a nitro group interacting with a hydrogen bonded to an oxygen or nitrogen, and (d) the oxygen of a nitro group interacting with the nitrogen of a nitro group on another molecule. The minimum *s*_*r*_ observed for each type of interaction is indicated by a dashed black line.

Energetic materials have nitrogen-containing groups that participate in unique interactions. Analysis of the CSD has been conducted using the ISOSTAR software^110^ to determine whether new default *s*_*r*_ values are needed for EMs. The results are presented in Figure 2. The minimum *s*_*r*_ found for NO_2_ hydrogen bonded to NH_2_ is 0.68. The minimum *s*_*r*_ found for NO_2_ interacting with hydrogen bonded to carbon is 0.80. The minimum *s*_*r*_ found for NO_2_ interacting with hydrogen bonded to oxygen or nitrogen via hydrogen bonds is 0.65. The minimum *s*_*r*_ found for NO_2_ interacting with NO_2_ is 0.90. Therefore, the *s*_*r*_ value of 0.6 for strong hydrogen bonds previously determined for Genarris 2.0 provides good coverage for NO_2_ groups. A new *s*_*r*_ value of 0.80 has been implemented for NO_2_ interacting with hydrogen bonded to carbon. All other interactions examined here are covered by the default *s*_*r*_ setting of 0.85.

For each target, a single Genarris run was conducted using an *s*_*r*_ of 0.85. Genarris obtains the estimated unit cell volume by multiplying the molecular solid-form volume obtained from PyMoVE^66^ by the number of molecules per cell. The volume distribution for structure generation is centered around the estimated volume with a standard deviation of 30 Å^3^. PyMoVE gave volume estimates of 774, 1193, 2132, and 532 Å^3^ for LLM-105, α-RDX, α-HMX, and β-HMX, respectively. Compared to the experimental volumes of 748, 1597, 2138, and 519 Å^3^, this results in errors of 3.43, 25.3, 0.29, and 2.5%, respectively. Raw pools were downselected to the final pool of structures using the Robust workflow as described briefly above and detailed in ref (68). The final structures were then optimized and checked for duplicates before being used as initial populations for GAtor. Plots of volume distributions, space group distributions, and lattice parameter distributions through the steps of the Genarris workflow are provided in the Supporting Information (SI).

### GAtor

GAtor provides the user with maximal flexibility by offering several options for performing various GA operations. Different options may be optimal for different systems, depending on the structure of the PES. Therefore, we recommend conducting several GAtor runs with different settings. Two fitness functions are available in GAtor: energy-based and evolutionary niching.^52,63^ The energy-based fitness function assigns higher fitness to lower-energy structures. However, this can lead the GA to exhibit “genetic drift”, i.e., the oversampling of wide, low-energy basins. The evolutionary niching fitness function is designed to combat genetic drift by penalizing the fitness of structures in oversampled portions of the PES, driving the GA toward sampling underrepresented regions. This is achieved by using AP to dynamically cluster the population and dividing the fitness of the structures belonging to a certain cluster by the number of structures in the cluster. Structures are selected for mating using one of two schemes: roulette wheel selection or tournament selection. In the roulette wheel selection scheme, fitter structures have larger slices of the wheel, increasing their probability of being selected. In the tournament selection scheme, a user-defined number of structures is randomly chosen from the population to compete in a tournament where the two structures with the highest fitness win. In GAtor, there are three breeding schemes for generating offspring: standard mutation, standard crossover, and symmetric crossover. The standard mutation scheme modifies one parent structure through random translations, strains, permutations, and rotations. Crossover schemes combine the genes of two parent structures with random weights. Random fractions of the lattice vectors of the parent structures are combined to form those of the child. In the standard crossover scheme, the Euler angles are computed to define the orientation of the molecules within the unit cell of both parent structures. The orientation of the molecules in the child structure is determined by randomly combining fractions of the parents’ Euler angles. In the symmetric crossover scheme, the child inherits its space group directly from one of the parents and the relevant space group symmetry operations are performed on the asymmetric unit to construct the child structure. The symmetric crossover scheme typically generates higher symmetry structures than the standard crossover scheme.^52^ For this work, all of the targets were run with three different settings using the evolutionary niching fitness function: 100% mutation (Mutation), 75% standard crossover probability (Standard), and 75% symmetric crossover probability (Symmetric). The tournament selection scheme was used for all of the GA runs with a tournament size of 10 structures.

### DFT Settings

Geometry relaxations and energy evaluations were performed using DFT, as implemented in the electronic structure code FHI-aims,^111^ which is interfaced with GAtor and Genarris. All DFT calculations performed within GAtor and Genarris used PBE+TS with *lower-level* numerical settings, which correspond to the light species default settings, with light integration grids and tier 1 basis sets. A 3 × 3 × 3 *k*-point grid was used to sample the Brillouin zone. No constraints were applied during unit cell optimization in both Genarris and GAtor, such that the lattice parameters, angles, and space group symmetry were allowed to change.

Reoptimization and reranking were performed using PBE+TS with the default intermediate species settings for the basis set and the integration grid.^112^ The intermediate basis sets have a reduced set of tier 2 radial functions. Rerelaxation with more stringent numerical settings and larger basis sets may cause some structures to relax to the same local minimum. Therefore, duplicates were detected and removed again at this point. Subsequently, the structures were rerelaxed and reranked using PBE+MBD with intermediate settings. After relaxations with PBE+MBD, duplicates were removed again and the remaining structures were rerelaxed and reranked using PBE+XDM. Following another round of duplicate removal, final reranking was performed for the remaining structures. Single point energy evaluations, using the PBE+MBD geometries, were performed using PBE0+MBD with intermediate numerical settings. A comparison of the intermediate settings to tier 2 settings with tight species defaults is provided in the SI.

Vibrational contributions were calculated using Phonopy,^113^ a python package that interfaces with FHI-aims. Phonopy uses the finite difference method to calculate vibrational effects within the harmonic approximation and uses FHI-aims to calculate the forces on displaced atoms. Supercells for phonon calculations were defined such that each lattice vector was at least 10 Å.^85,89,95,107^ Phonon calculations were performed on geometries optimized with PBE+MBD using intermediate settings. A *k*-grid of 3 × 3 × 3 was used to perform SPE calculations for each displaced atom with PBE+MBD using the same settings as described above. The vibrational energy contribution displayed in the main text was evaluated at the crystallization temperature of each target, 294, 295, 401, and 295 K for LLM-105, α-RDX, α-HMX, and β-HMX, respectively. Temperature-dependent ranking plots are provided in the SI.

## Results and Discussion

### LLM-105

**Figure 3:**
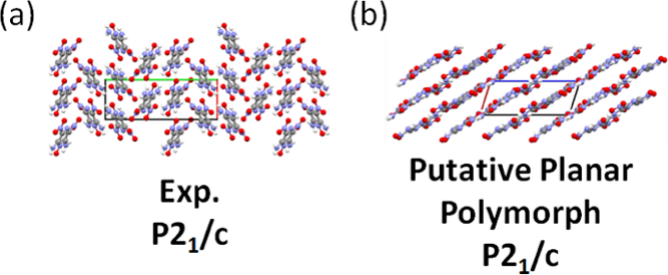
(a) Experimental structure of LLM-105 and (b) a putative planar polymorph generated by GAtor. The *a*, *b*, and *c* lattice vectors are shown in red, blue, and green, respectively.

The LLM-105 molecule has two nitro (NO_2_) and two amine (NH_2_) groups. The only known crystal structure of LLM, illustrated in Figure 3a, crystallizes in the space group *P*2_1_/*c*, has four molecules in the unit cell, and exhibits a herringbone packing motif.^91^ There is interest in identifying and synthesizing layered polymorphs of energetic materials because planar packing motifs are thought to be correlated with lower sensitivity.^114−117^

**Figure 4:**
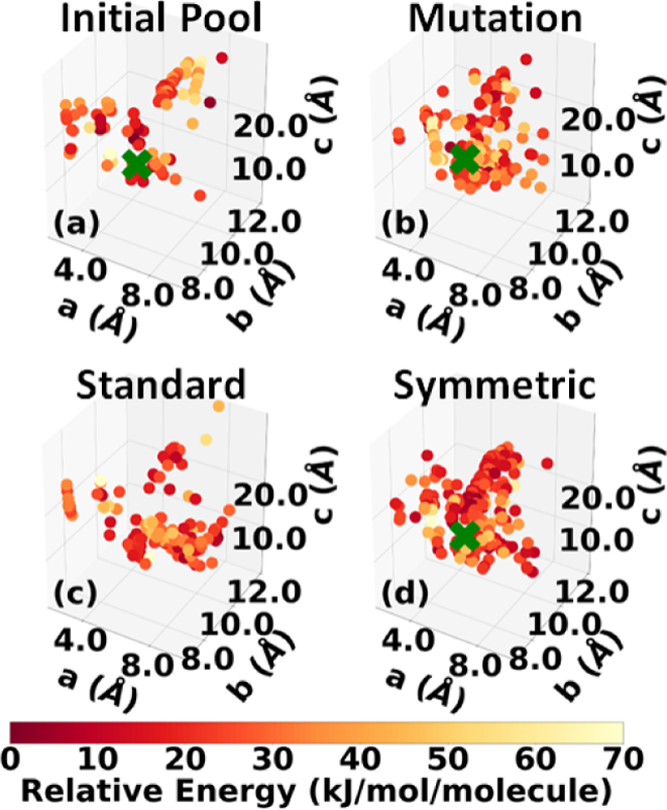
Lattice parameter distributions of the LLM-105 structures in (a) the initial pool generated by Genarris and the final populations generated by GAtor using (b) mutation-only, (c) standard crossover, and (d) symmetric crossover. The experimental structure is indicated by a green cross if found.

For this target, Genarris generated a raw pool of 20,000 structures, which was downselected to 106 structures. Figure 4a shows the lattice parameter distribution of the final pool of relaxed structures. Genarris successfully generated the experimental structure, as indicated by the green cross (an enlarged plot is provided in the SI). The region around the experimental structure is well-represented in the initial pool. The experimental structure was removed from the initial population to assess GAtor’s ability to generate it.

**Figure 5:**
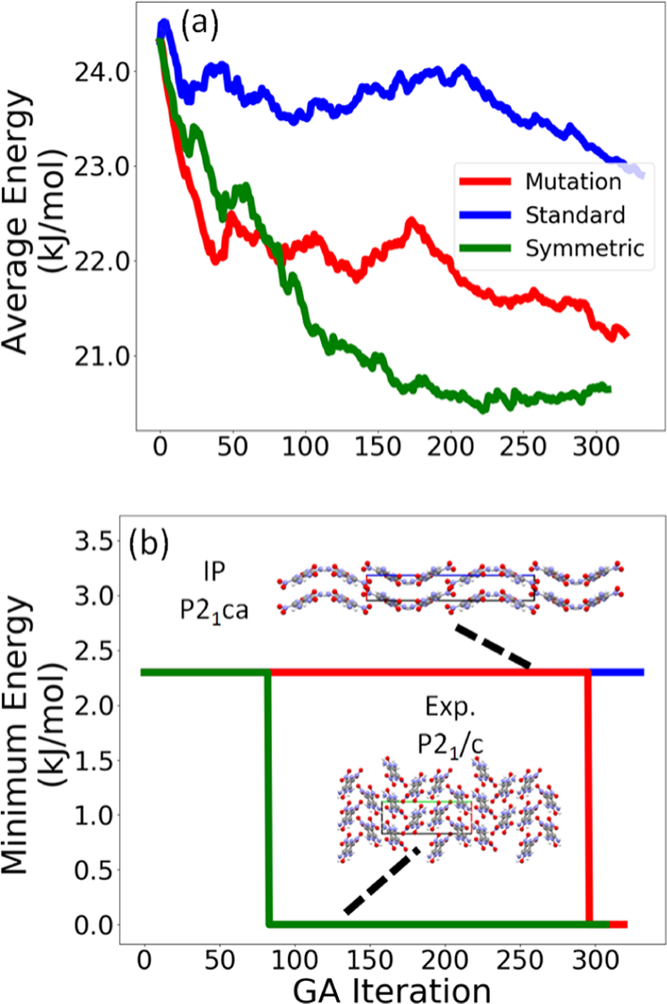
Relative average energy (a) and minimum energy (b) as a function of GA iteration for the three GA runs for LLM-105. The packing motif of the experimental structure, which was identified as the minimum energy structure, is shown as well as the minimum energy structure for the GA run using standard crossover. The *a*, *b*, and *c* lattice vectors are shown in red, green, and blue, respectively.

Figure 5 shows the average energy and minimum energy of the population relative to the lowest energy structure found in all of the GA runs as a function of GA iteration for the three GA runs conducted for LLM-105. Because a GA is not guaranteed to reach the global minimum, the average energy of the population of structures can be used as the convergence criterion. When the average energy is no longer decreasing, the GA run may be considered saturated. Alternatively, a GA run may be stopped after a certain number of cycles (300 in this case). The GA run using symmetric crossover displays the smoothest convergence behavior, with the average energy saturating at around 21 kJ/mol. This run generates the experimental structure the fastest at iteration 83. The GA run using only mutations has a slightly more erratic convergence behavior, but it too converges around 21 kJ/mol. This run generates the experimental structure at iteration 296. The GA run using standard crossover converges to a higher average relative energy value of 23 kJ/mol and fails to generate the experimental structure. The minimum energy structure for this run is an initial pool structure that exhibits a β sheet packing motif, as shown in Figure 5b.

Figure 4b–d shows the lattice parameter distributions of structures generated by the GA during the three GAtor runs (an enlarged plot is displayed in the SI). This can provide insights into how different GA settings explore the landscape of lattice parameters, as well as the overall structure of the PES. The lattice parameter space of LLM-105 is not characterized by distinct basins but rather a continuous well along the *b* lattice vector. The experimental structure resides in a portion of the lattice parameter space with shorter *a* lattice vectors. The mutation and symmetric crossover schemes sample this region more than the standard crossover scheme. The run using the standard crossover scheme explores the lattice parameter space less extensively than the other two runs and remains trapped in one region. This could explain why the standard crossover scheme fails to generate the experimental structure. Typically, the standard crossover scheme tends to generate lower symmetry structures. This is detrimental to LLM-105.

**Figure 6:**
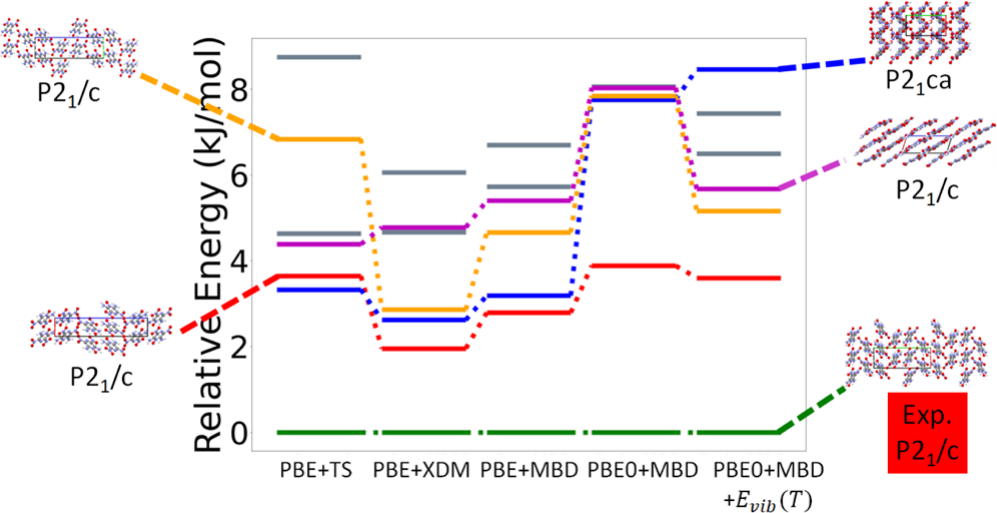
Hierarchical reranking of LLM-105 structures generated by GAtor using increasingly accurate dispersion-inclusive DFT methods. Relative energies are referenced to the lowest energy structure with each method. The packing motifs of the experimental structure and other structures of interest are shown with the *a*, *b*, and *c* lattice vectors colored in red, green, and blue, respectively.

Figure 6 shows the hierarchical reranking using increasingly accurate DFT functionals and dispersion methods. The experimental structure, shown in green, is consistently ranked as the lowest energy structure. At every level of theory, there is a gap of 2–4 kJ/mol between the experimental structure and other putative structures. The structure shown in red with a herringbone packing motif is ranked as the second most stable by all methods except for PBE+TS. The ranking of other low-lying structures changes depending on the method used. The herringbone structure shown in orange is stabilized by MBD compared to TS, and even more so by XDM. This structure, the herringbone structure shown in blue, and the layered structure shown in purple (also shown in Figure 3b) are significantly destabilized when switching from PBE to PBE0. The vibrational contribution stabilizes the structures shown in orange and purple but destabilizes the structure shown in blue. With PBE0+MBD+*E*_vib_(*T*), the layered structure shown in purple is about 6 kJ/mol higher in energy than the experimental structure. It has been found that non-conformational polymorphs are typically within 4 kJ/mol or less of each other, but larger energy differences, up to 10 kJ/mol, have been observed in some cases.^7,118^ Therefore, the putative layered structure could be within the polymorph range. Metastable polymorphs may become thermodynamically stable at higher temperatures or pressures or be kinetically stabilized by changing the growth conditions.^57,119,120^ Additional rankings up to 500 K are shown in the Figure SI.

**Figure 7:**
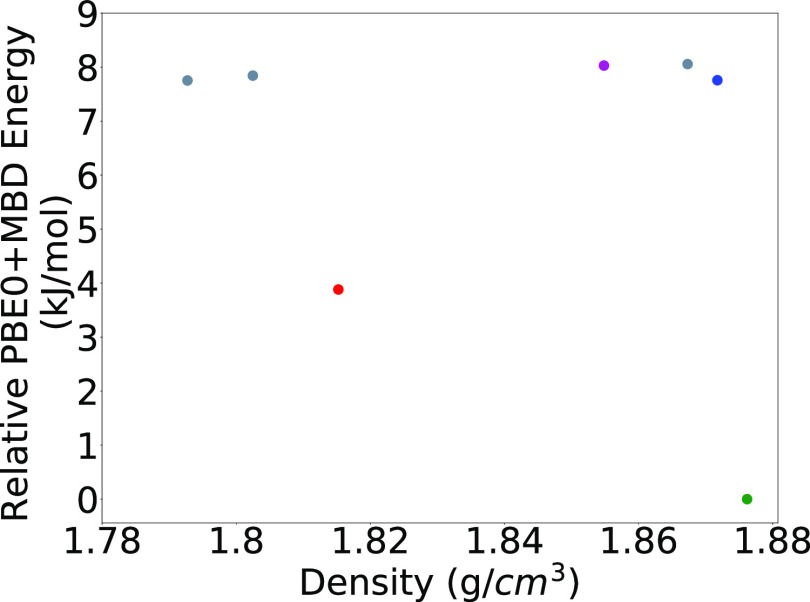
PBE0+MBD relative energy as a function of density for LLM-105 structures generated by GAtor. Colored markers correspond to the structures shown in Figure 6.

Density is a key property in EMs. Figure 7 shows the relationship between the relative PBE0+MBD energy and the density of the lowest energy LLM-105 structures generated by GAtor (the relative energy, as obtained with PBE+TS and *lower-level* settings within the GA, is plotted as a function of density for all of the generated structures in the SI). The structures shown in color are the same as in Figure 6. The experimental structure, colored in green, is the most dense. However, density is not strongly correlated with stability. For example, several putative structures are more dense but less stable than the structure colored in red, which was generated by the run using symmetric crossover. Interestingly, the putative planar polymorph, colored in purple, is only slightly less dense than the experimental structure. This structure is in a cluster of dense, less-stable structures, which includes the blue structure. The purple structure was generated by the run using mutation-only, and the other two structures in the cluster are from the initial pool. The two remaining structures, in the top-left portion of Figure 7, are also initial pool structures.

### α-RDX

The RDX molecule has alternating nitro and methyl side-groups. The six-membered ring of RDX is nonplanar and buckled. RDX has several known polymorphs. The most stable form, α-RDX, has eight molecules in the unit cell and crystallizes in the space group *Pbca*.^121^ The metastable form β-RDX has been grown and characterized in ambient conditions.^9,29,122,123^ The β form has eight molecules per unit cell and crystallizes with two molecules in the asymmetric unit in the space group *Pca*2_1_.^122^ In addition, RDX has three known high-pressure forms: γ, δ, and ϵ. The γ polymorph has eight molecules per unit cell with two molecules in the asymmetric unit in the space group *Pca*2_1_.^27^ The crystal structure of the δ polymorph has not been determined, though Raman spectroscopy results suggest that the β and δ forms have similar molecular symmetry and crystal structures.^26,124,125^ The ϵ polymorph has four molecules per unit cell and crystallizes in the space group *Pca*2_1_.^28^ The different forms of RDX are conformational polymorphs. In the α form, two of the three nitro groups adopt an axial conformation and the third adopts an equatorial conformation, as shown in Figure 1. In the β form, all three nitro groups adopt an axial conformation. In the γ form, there are two independent molecules in the asymmetric unit, one of which adopts the same conformation as the α form and one of which has two nitro groups in the equatorial position and one nitro group in the axial position.^27^ The molecules in the ϵ polymorph have all three nitro groups in the axial position.^28^

**Figure 8:**
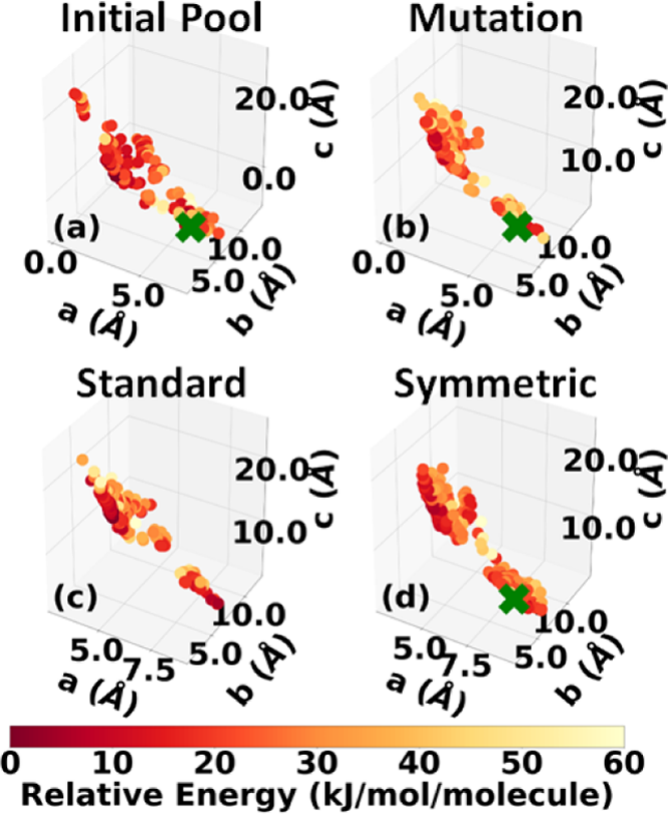
Lattice parameter distributions of the α-RDX structures in (a) the initial pool generated by Genarris and the final populations generated by GAtor using (b) mutation-only, (c) standard crossover, and (d) symmetric crossover. The experimental structure is indicated by a green cross if found.

The conformation of α-RDX was used to generate the initial pool with Genarris. For α-RDX, Genarris generated a raw pool of 20,000 structures, which was downselected to 131 structures. Figure 8a shows the lattice parameter distribution of the final relaxed structures (an enlarged plot is provided in the SI). The experimental structure was generated, as indicated by the green cross. In addition to sampling the basin of the experimental structure, Genarris explored diverse, low-energy portions of the PES, which is key to cultivating a good initial population for GAtor. The experimental structure was removed from the initial population to assess GAtor’s ability to generate it.

**Figure 9:**
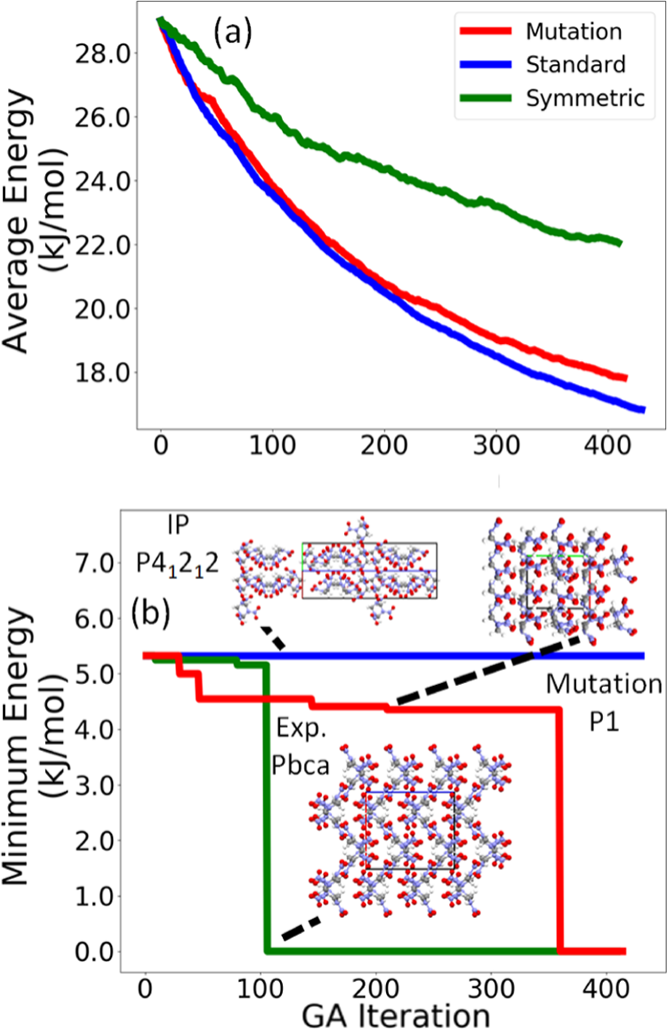
Relative average energy (a) and minimum energy (b) as a function of GA iteration for the three GA runs for α-RDX. The packing motif of the experimental structure, which was identified as the minimum energy structure, as well as the minimum energy structure of the run using standard crossover and an intermediate structure generated by the run using mutation-only, are shown. The *a*, *b*, and *c* lattice vectors are shown in red, green, and blue, respectively.

Figure 9 shows the average energy and minimum energy of the population relative to the lowest energy structure found in all of the GA runs as a function of GA iteration for the three GA runs conducted for α-RDX. In contrast to LLM-105, in this case, all three runs converge smoothly, with the average energy decreasing monotonically. The runs using standard crossover and mutation-only converge to around 18 kJ/mol above the global minimum, whereas the run using symmetric crossover converges to about 24 kJ/mol. We note that the average energy of the population depends on the regions of the PES being explored and is not necessarily correlated with the minimum energy. The experimental structure is generated by the run using symmetric crossover after 106 iterations and by the run using mutation-only after 360 iterations. Similar to LLM-105, the run using standard crossover fails to generate the experimental structure of α-RDX. The minimum energy structure for this run is an initial pool structure, whose packing motif is shown in Figure 9b. Interestingly, this structure packs in a highly symmetric space group (*P*4_1_2_1_2). However, the run using mutation-only finds a rather low-energy structure that packs in the lowest symmetry space group (*P*1) before generating the experimental structure. This structure, unlike the initial pool structure, has a similar packing motif to the experimental structure.

**Figure 10:**
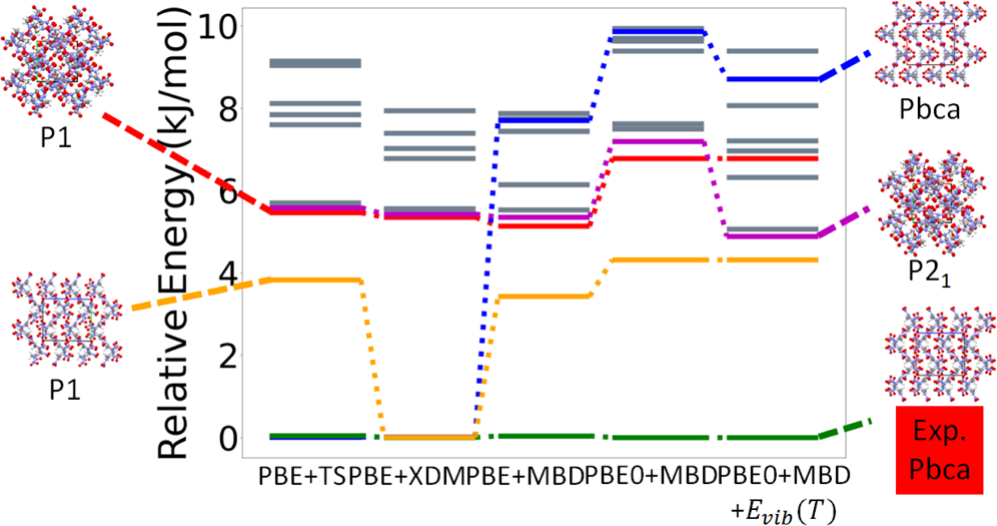
Hierarchical reranking of α-RDX structures generated by GAtor using increasingly accurate DFT methods. Relative energies are referenced to the lowest energy structure with each method. The experimental structure and other low-energy structures are illustrated with the *a*, *b*, and *c* lattice vectors shown in red, green, and blue, respectively.

Figure 8b–d shows the lattice parameter distributions of structures generated by the three GAtor runs (an enlarged plot is provided in the SI). The PES of α-RDX is characterized by two basins. One basin, where the experimental structure resides, is in the bottom-right portion of the lattice parameter plot with longer *a* lattice parameters and shorter *c* lattice parameters. This basin contains both high- and low-energy high-symmetry structures with packing motifs similar to the experimental structure. The run using symmetric crossover yields the best sampling of this portion of the PES, followed by the run using mutation only. The symmetric crossover typically produces higher symmetry structures. This explains the relatively fast generation of the experimental structure in the GA run using symmetric crossover. The other basin, in the top-left portion of the lattice parameter plot, with shorter *a* lattice parameters and longer *c* lattice parameters, contains both high- and low-energy structures with a denser packing motif than the experimental structure. This basin generally contains low symmetry structures. An example of one of these structures is shown in red in Figure 10. The run using standard crossover samples more from the basin in the top left of the lattice parameter distribution than from the basin of the experimental structure, which may explain its failure to generate the experimental structure.

Figure 10 shows the results of hierarchical reranking with increasingly accurate DFT methods for α-RDX. The experimental structure, shown in green, is consistently ranked as the lowest energy structure with all methods used here. The ranking of two putative structures, shown in blue and orange, changes significantly depending on the method. The structure shown in blue is ranked as nearly degenerate with the experimental with both pairwise dispersion methods, PBE+TS and PBE+XDM. When switching to MBD, and from PBE to PBE0, this structure is significantly destabilized to about 8–10 kJ/mol above the experimental structure. The structure shown in orange is ranked as nearly degenerate with the experimental structure with PBE+XDM. With all other methods, it is ranked as the second most stable structure and is about 4–4.5 kJ/mol higher in energy than the experimental structure. The structures shown in red and purple are ranked as very close in energy by the PBE functional with all dispersion methods. Both structures are destabilized when switching from PBE to PBE0, with the red structure remaining 1 kJ/mol more stable than the purple structure. When vibrational contributions are considered, however, the structure shown in purple is significantly stabilized, by about 2 kJ/mol, in comparison to the structure shown in red.

**Figure 11:**
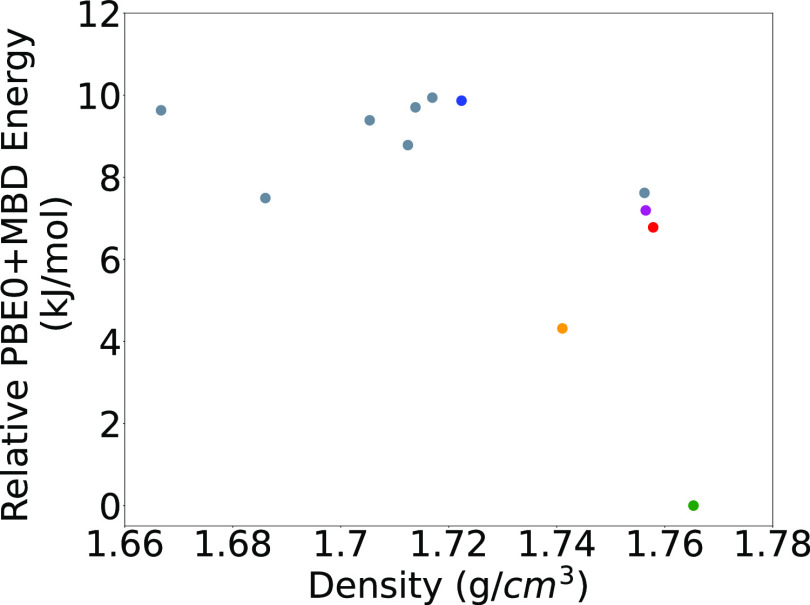
PBE0+MBD relative energy as a function of density for α-RDX structures generated by GAtor. Colored markers correspond to the structures shown in Figure 10.

Figure 11 shows the relative PBE0+MBD energy of the low-energy structures generated by GAtor as a function of density (the relative energy, as obtained with PBE+TS and *lower-level* settings within the GA, is plotted as a function of density for all of the generated structures in the SI). The experimental structure, shown in green, is the most dense structure. For α-RDX, density is somewhat correlated with stability. The cluster with the structure colored in red contains structures with relatively high density, all of which reside in the top-left region of the lattice parameter plots in Figure 8. The structure shown in red was generated by the GA run using symmetric crossover, whereas the other two structures in the cluster were generated by the runs using standard crossover and mutations only. The other relatively dense, low-energy structure, shown in orange, was generated by the GA run using only mutations and resides in the same region of the lattice parameter space as the experimental structure in Figure 8. This demonstrates the importance of performing several GA runs with different settings for achieving a more thorough sampling of the PES.

### HMX

HMX has a nonplanar eight-membered alternating carbon and nitrogen ring with alternating nitro and methyl side-groups. HMX has several known polymorphs. The β form is the most stable polymorph at ambient conditions^126−129^ and has the lowest shock sensitivity. The α form is stable from 103 to 162 °C.^126^ The β-HMX phase has two molecules per unit cell and crystallizes in the space group *P*2_1_/*c*, and the α form has eight molecules per unit cell and crystallizes in the space group *Fdd*2.^126^ HMX also has another high-temperature form, δ, which has six molecules per unit cell and crystallizes in the space group P6_1_.^13^ The γ form is a hemihydrate with four molecules per unit cell and crystallizes in the space group *P*2/*c*.^12,14^ The β and ϵ forms adopt a chair conformation, while the α, γ, and δ forms adopt a boat conformation.^13,14,126^ To demonstrate the ability of Genarris and GAtor to account for conformational polymorphism, we perform CSP for the α and β polymorphs. The α phase is the largest system investigated here, with 224 atoms in the unit cell. Although, in principle, a structure with a *Z* = 2 could be generated in a *Z* = 8 search as a supercell, the β form is not expected to be found in the search for the α form because it comprises a different conformer.

**Figure 12:**
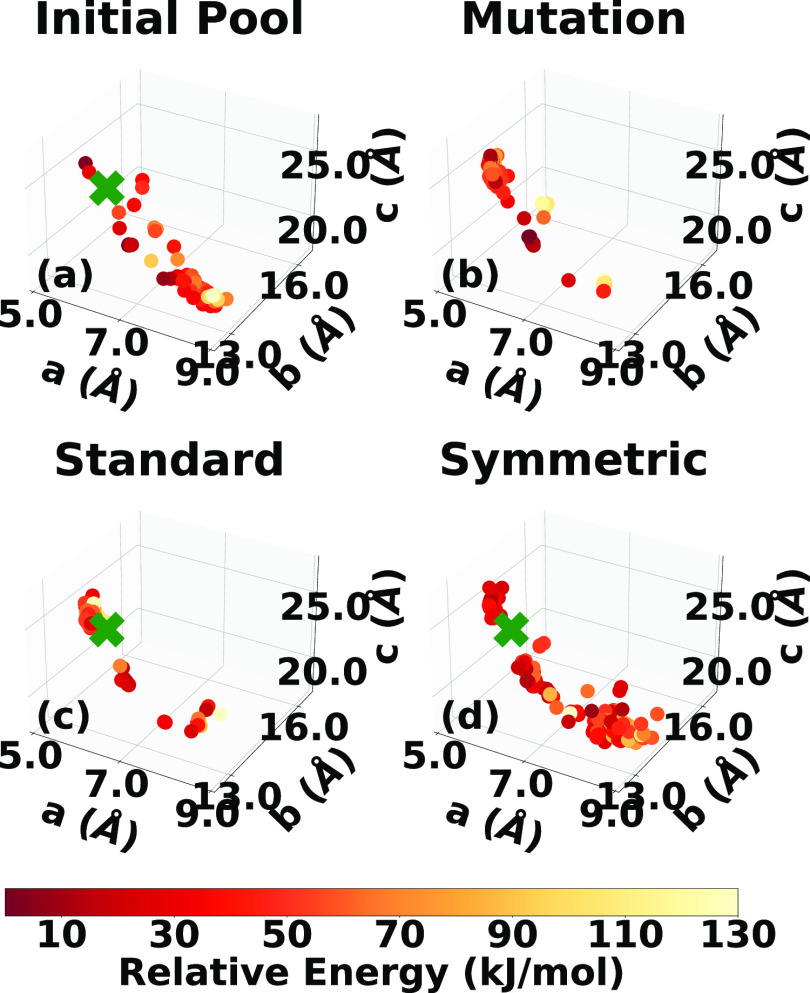
Lattice parameter distributions of the α-HMX structures in (a) the initial pool generated by Genarris and the final populations generated by GAtor using (b) mutation-only, (c) standard crossover, and (d) symmetric crossover. The experimental structure is indicated with a green cross if found.

**Figure 13:**
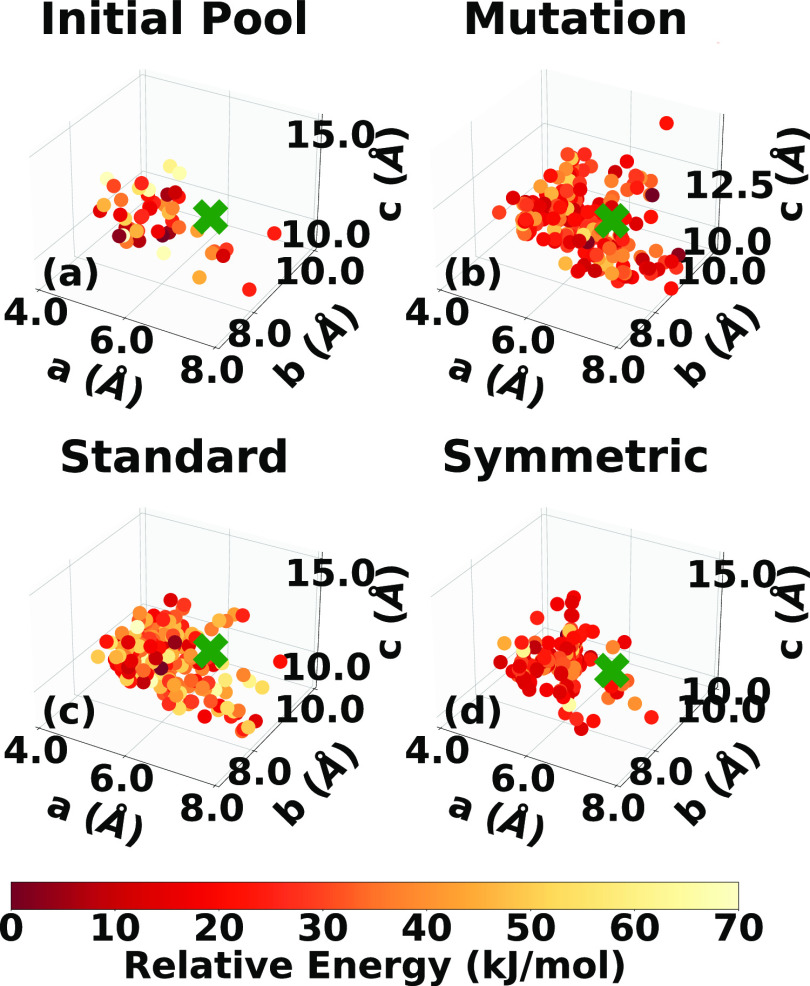
Lattice parameter distributions of the β-HMX structures in (a) the initial pool generated by Genarris and the final populations generated by GAtor using (b) mutation-only, (c) standard crossover, and (d) symmetric crossover. The experimental structure is indicated by a green cross if found.

Genarris generated raw pools of 11,400 and 10,000 structures for α-HMX and β-HMX, respectively, which were then downselected to 52 and 50 structures, respectively. Figures 12a and 13a show the lattice parameter distributions of the final pool of relaxed structures for α-HMX and β-HMX, respectively (enlarged views of both plots are provided in the SI). Genarris successfully generated the experimental structure for both polymorphs, as indicated by the green cross. The experimental structure was removed from the initial population to assess GAtor’s ability to generate it.

**Figure 14:**
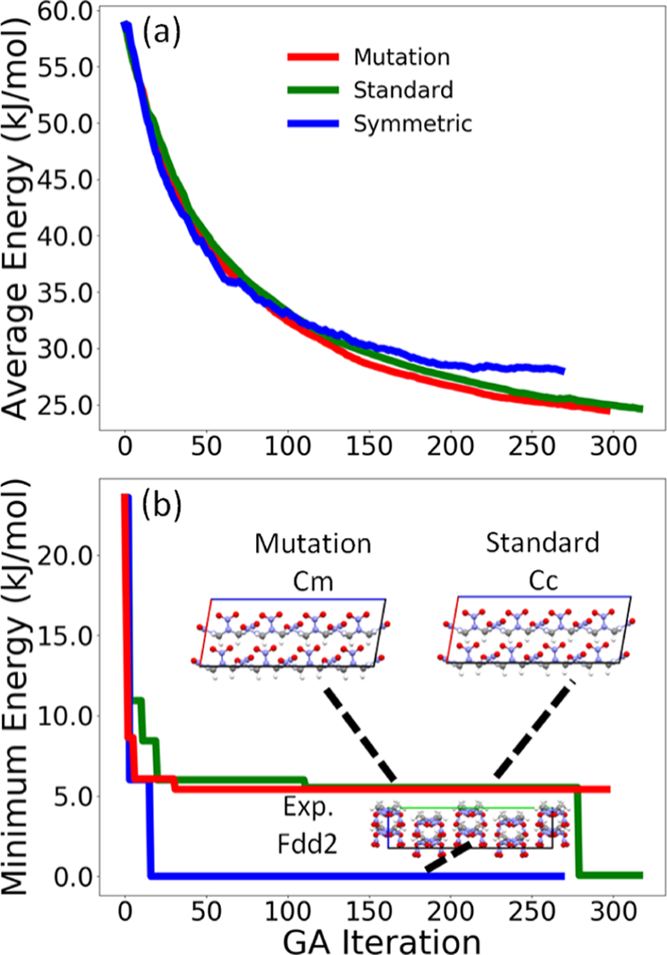
Relative average energy (a) and minimum energy (b) as a function of GA iteration for the three GA runs for α-HMX. The packing motifs of the experimental structure, as well as low-energy structures, generated by the runs using standard crossover and mutation-only, are also shown. The *a*, *b*, and *c* lattice vectors are shown in red, green, and blue, respectively.

**Figure 15:**
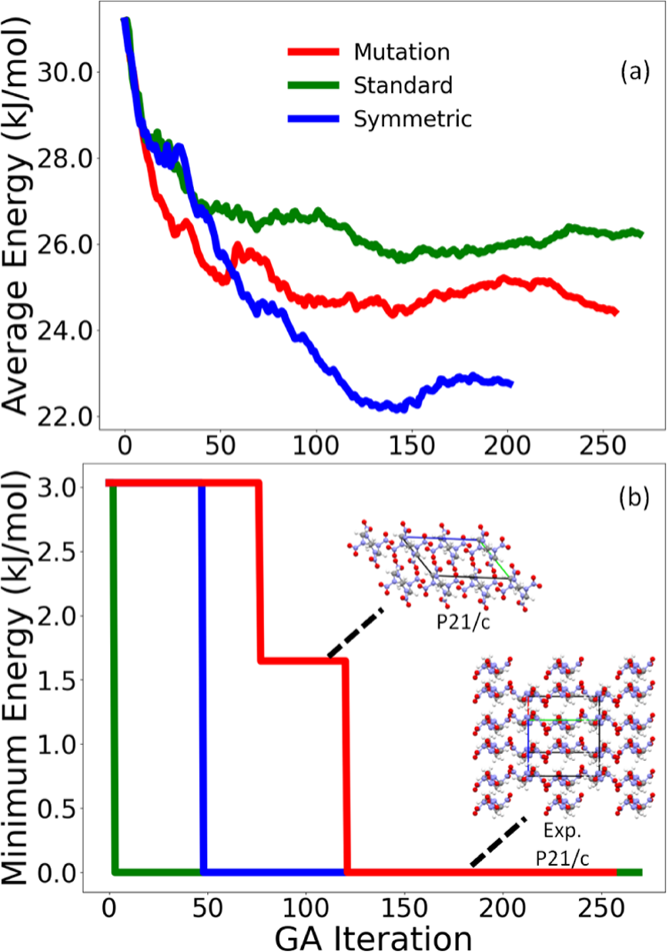
Relative average energy (a) and minimum energy (b) as a function of GA iteration for the three GA runs for β-HMX. The packing motif of the experimental structure, as well as low-energy structures generated by the run using mutation-only, are also shown. The *a*, *b*, and *c* lattice vectors are shown in red, green, and blue, respectively.

Figures 14 and 15 show the average energy and minimum energy of the population relative to the lowest energy structure found in all of the GA runs as a function of GA iteration of the three GA runs conducted for α-HMX and β-HMX, respectively. For both forms of HMX, all three runs converge smoothly, with the average energy decreasing monotonically to an average energy value of around 25 kJ/mol. For α-HMX, the symmetric crossover run is the fastest to generate the experimental structure, at iteration 16, as seen in Figure 14b. The run using standard crossover generates the experimental structure at iteration 279. The minimum energy structure prior to the generation of the experimental structure packs in the lower-symmetry *Cc* space group. The mutation-only run fails to generate the experimental structure of α-HMX. The minimum energy structure generated by this run is still around 5 kJ/mol higher in energy than the experimental structure and packs in a lower symmetry space group, *Cm*. The runs using standard crossover and mutation-only struggle to generate higher symmetry structures, which could explain why the experimental structure of α-HMX takes many iterations to generate or is not generated at all in these runs. For β-HMX, all three GAtor runs generate the experimental structure, as seen in Figure 15b. The standard crossover scheme generates the experimental structure the fastest at iteration 3, followed by symmetric crossover at iteration 48, and mutation at iteration 121. In the mutation-only run, the minimum energy structure prior to the generation of the experimental structure packs in the same space group as the experimental structure but has a different packing motif. HMX is the only target for which the standard crossover scheme successfully generates the experimental structures.

**Figure 16:**
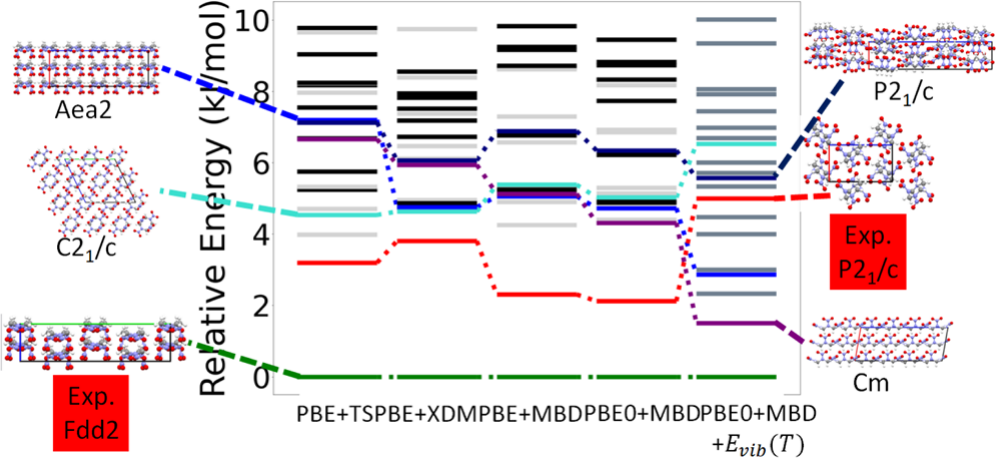
Hierarchical reranking of HMX structures generated by GAtor using different dispersion-inclusive DFT methods. Relative energies are referenced to the lowest energy structure with each method. Cooler colors and gray lines correspond to structures that have the α conformer, while hotter colors and black lines correspond to structures that have the β conformer. The experimental structure and other low-energy structures are illustrated with the *a*, *b*, and *c* lattice vectors shown in red, green, and blue, respectively.

Figures 12b–d and 13b–d show the lattice parameter distributions of structures generated by the three GAtor runs for α-HMX and β-HMX, respectively. For α-HMX, the PES is characterized by a wide basin with relatively long *a* lattice parameters and relatively short *c* parameters in the bottom-right portion and a scattering of narrower basins, with relatively shorter *a* parameters and relatively long *c* parameters in the top-left portion. The experimental structure resides in the latter region. Structures in this top-left portion exhibit a similar packing motif to the experimental structure but with a lower symmetry. Some of these structures are shown in Figure 16. The high symmetry of the experimental structure explains why the symmetric crossover run is able to generate it fast despite lower sampling in this region of the PES. The symmetric crossover run heavily samples the bottom-right portion of the PES, which contains structures with a packing motif similar to the structure shown in blue in Figure 16. Structures in this region of the PES tend to pack in low symmetry space groups, including those generated by the symmetric crossover scheme. For β-HMX, the PES is not characterized by distinct basins, similar to LLM-105. Even though the mutation-only run samples the most from the area of the PES where the experimental structure resides, it takes the longest to generate it. This is because lattice parameters close to the experimental structure do not necessarily correlate with similar packing motifs. The two crossover schemes sample mostly from areas with shorter *a* lattice vectors compared to the experimental structure. For both crossover schemes, the experimental structure appears to reside at the edge of the PES region sampled. The standard crossover samples more high-energy structures than either the mutation-only and symmetric crossover runs, suggesting that the quick generation of the experimental structure could be fortuitous.

Figure 16 shows the results of hierarchical reranking with increasingly accurate DFT methods for HMX. The experimental structure for α-HMX, shown in green, is the most stable at every level of theory. The experimental structure for β-HMX, shown in red, is ranked 3 kJ/mol above the α form with PBE+TS, destabilized to 4 kJ/mol with PBE+XDM, and then stabilized with PBE/0+MBD to 2 kJ/mol above the α form. A previous study obtained similar results using other dispersion-inclusive DFT methods.^10^ Vibrational contributions destabilize the β form to 5 kJ/mol above the α form. Even when we account for thermal expansion within the quasi-harmonic approximation, the β form is still about 2 kJ/mol higher in energy than the α form (see the Supporting Information). It is possible that the discrepancy between our results and experimental observations is due to the insufficient accuracy of dispersion-inclusive DFT methods.^89^ We note, however, that there has been some ambiguity in the literature regarding the transition from β to α,^128−132^ which has never been studied in the absence of a solvent. Brill et al. have suggested that kinetic factors rather than thermodynamic enthalpy changes play a significant role in the stabilization and conversion between HMX polymorphs.^128^ Others have discussed the large uncertainty in true values for enthalpy changes in the HMX polymorphs,^130−132^ which may be indicative of kinetic effects that arise because of the strong nucleation and growth barriers between HMX polymorphs.^129^ Ranking with an explicit consideration of thermal expansion calculated within the quasi-harmonic approximation is available in the SI.

Of the other putative structures generated by GAtor, the structure shown in purple is stabilized with increasing levels of theory, with a final ranking of 2 kJ/mol above the α form with PBE0+MBD+*E*_vib_(*T*). The structures shown in navy and blue are ranked as nearly degenerate with PBE+TS. The blue structure is stabilized compared to the navy structure with PBE+XDM, PBE+MBD, and PBE0+MBD. Accounting for vibrational contributions in PBE0+MBD+*E*_vib_(*T*) further stabilizes the blue structure, placing it 3-4 kJ/mol above the α form, whereas the navy structure remains 6 kJ/mol above the α form. The structure shown in cyan is ranked roughly the same with all methods, with the exception of a significant destabilization when vibrational contributions are considered. This demonstrates the need for hierarchical reranking using the most accurate methods and for considering vibrational contributions.

**Figure 17:**
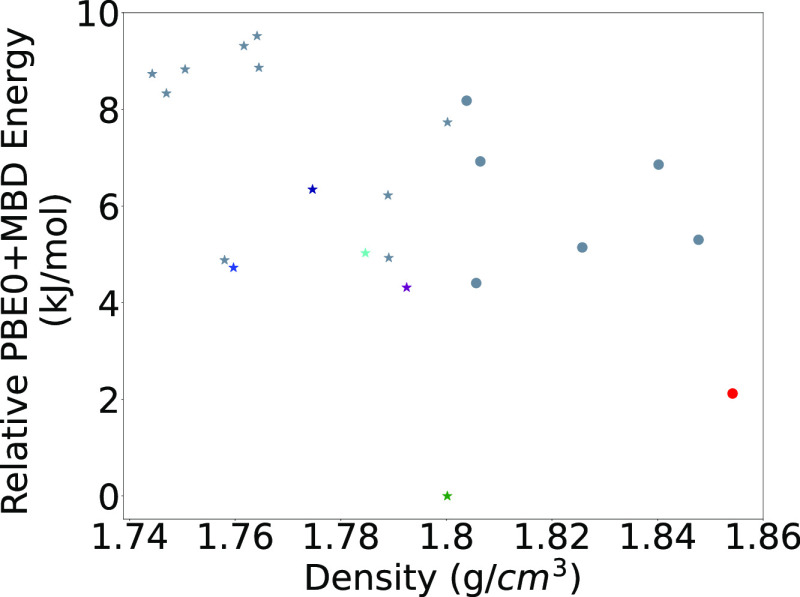
PBE0+MBD relative energy as a function of density for HMX structures generated by GAtor. Round markers indicate structures with the β conformer, and star markers indicate structures with the α conformer. Colored markers correspond to the structures shown in Figure 16.

Figure 17 shows the relative PBE0+MBD energy of the low-energy structures generated by GAtor as a function of density. The experimental structures of α-HMX and β-HMX are shown in green and red, respectively. The β form is the most dense, in agreement with the literature.^126^ Several structures have a similar density to the α form. For HMX, density is somewhat correlated with stability, with the α form being an outlier. The structure shown in cyan resides in the bottom-right portion of the lattice parameter plots in Figure 12. The other structures with similar density reside in the top-left portion of the lattice parameter plots. Four of the structures in this dense cluster were generated using crossover, two by each scheme, while the remaining structure was generated by mutation. This demonstrates the importance of conducting several GA runs with different settings.

## Conclusion

In summary, we have conducted crystal structure prediction using the genetic algorithm, GAtor, and its associated structure generator, Genarris, for the energetic materials LLM-105 and α-RDX as well as the α and β forms of HMX, which are conformational polymorphs. The close-contact settings in Genarris were updated for NO_2_···CH interactions, common in EMs. For each target, three GAtor runs were performed using different settings for mating: mutation-only, standard crossover, and symmetric crossover, both with 75% probability. All three runs used the evolutionary niching feature of GAtor.

Both Genarris and GAtor successfully generated the experimental structures of all targets. The symmetric crossover scheme was the most successful in consistently generating the experimental structures of all materials, despite the differences between their potential energy landscapes. We note, however, that the three GAtor runs sampled different regions of the PES, and some of the lower-energy putative structures were only generated by the runs using standard crossover and mutation-only. This demonstrates the importance of conducting several GAtor runs with different settings to achieve diverse samplings of the PES. We have also demonstrated the ability of Genarris and GAtor to handle conformational polymorphism by generating both the α and β polymorphs of HMX in independently seeded calculations.

For LLM-105 and α-RDX, the experimental structures were ranked as the most stable with all dispersion-inclusive density functional theory methods used here. For HMX, the α form was persistently ranked as more stable than the β form, in contrast to experimental observations. This may be attributed to the insufficient accuracy of dispersion-inclusive DFT methods or possibly to kinetic effects that are unaccounted for in our calculations. Further investigation of the polymorphism of HMX is outside the scope of this work.

For all targets, the ranking of some putative structures changed significantly when switching from the pairwise methods to the many-body dispersion method, upon switching from the semilocal PBE functional to the PBE0 hybrid functional, and/or upon adding vibrational contributions. For HMX, when vibrational contributions were considered, several structures with the α conformer became more stable than the experimental β form. This is consistent with our past observations that some interactions and packing motifs are more sensitive than others to the method used.^89,92^ This demonstrates the importance of performing hierarchical reranking using increasingly accurate methods.

For all three materials, the experimental structure was found to be the most dense, a key property for EMs. However, density was not found to be strongly correlated with stability, as some putative structures, whose densities were close to the experimental structures, were predicted to be significantly less stable. For LLM-105, a putative polymorph with a planar packing motif, which is desirable thanks to its association with lower sensitivity, was generated by GAtor. This structure was about 6 kJ/mol higher in energy than the experimental structure with PBE0+MBD+*E*_vib_(*T*), which is at the edge of the typical energy window between non-conformational polymorphs.^7,118^ We note that metastable polymorphs may be possible to crystallize, e.g., by changing the growth conditions.^57,119,120^

In conclusion, we have demonstrated the ability of Genarris and GAtor to generate the experimental structures for three energetic materials. We have also shown that CSP can be used to identify putative polymorphs with desirable properties, such as the layered structure of LLM-105. More broadly, this work shows how computational CSP can guide experimental efforts in the field of energetic materials. CSP has already become an integral part of the pharmaceutical development process to help mitigate the risk of the appearance of unintended polymorphs. Similarly, we envision CSP becoming an integral part of the EM development pipeline. CSP may help discover putative crystal forms with desirable packing motifs and morphologies, complement X-ray diffraction experiments for high-pressure forms that are difficult to characterize, and inform equation-of-state models.

## Supporting Information Available

Benchmark of intermediate vs tight settings with FHI-aims; space group, volume, and lattice parameter distributions for each step of the Robust workflow in Genarris for each target; full energy-density plots and enlarged lattice parameter distribution plots for the generated structures of each target; computational cost of PBE+TS optimizations in GAtor; interaction chain analysis of structures whose ranking changes significantly with different methods for each target; and ranking of low-energy structures with vibrational corrections as a function of temperature (PDF)
Computed geometries of the crystal structures generated here (ZIP)
