# Supplementary material for: Ab Initio Crystal Structure Prediction of the Energetic Materials LLM-105, RDX, and HMX
Source: Cryst Growth Des. 2023 Aug 17;23(9):6275–89. doi: 10.1021/acs.cgd.3c00027 (PMC10763925; doi:10.1021/acs.cgd.3c00027)
Supplement: Supplementary file 1 — cg3c00027_si_001.pdf [file cg3c00027_si_001.pdf]

Supporting Information for: Ab Initio Crystal Structure Prediction of the Energetic Materials LLM-105, RDX, and HMX

Dana O'Connor[1], Imanuel Bier[1], Rithwik Tom[2], Anna M. Hiszpanski[3], Bradley A. Steele[3], and Noa Marom[1, 2,4]

[1]Department of Materials Science and Engineering, Carnegie Mellon University, Pittsburgh, PA, 15213, USA.
[2]Department of Physics, Carnegie Mellon University, Pittsburgh, PA, 15213, USA.
[3]Lawrence Livermore National Laboratory, Materials Science Division, Livermore, California 94550, USA
[4]Department of Chemistry, Carnegie Mellon University, Pittsburgh, PA, 15213, USA.

1 Intermediate Settings Benchmark in FHI-aims

In our previous work, we have used *higher-level* settings corresponding to the tier 2 settings and tight species defaults in FHI-aims for reoptimization and re-ranking in the post-processing stage of our CSP workflow.[1] In this work, we used the intermediate settings in FHI-aims.[2] Figure S1 shows four structures for LLM-105 calculated with the generalized gradient approximation of Perdew, Burke, and Ernzerhof (PBE)[3] with the Tkatchenko Scheffler pairwise dispersion method (PBE+TS),[4] and with the many-body dispersion method (PBE+MBD)[5,6] using *higher-level* and intermediate settings in FHI-aims. Due to the high computational expense and memory requirements, calculations using the PBE-based hybrid functional PBE0[7] paired with the MBD method were not possible at this time. As can be seen in Figure S1, the *higher-level* settings lowers the relative energy of most of the structures with both PBE+TS and PBE+MBD. The relative rankings do not change with PBE+MBD and two structures switch ranks with PBE+TS.

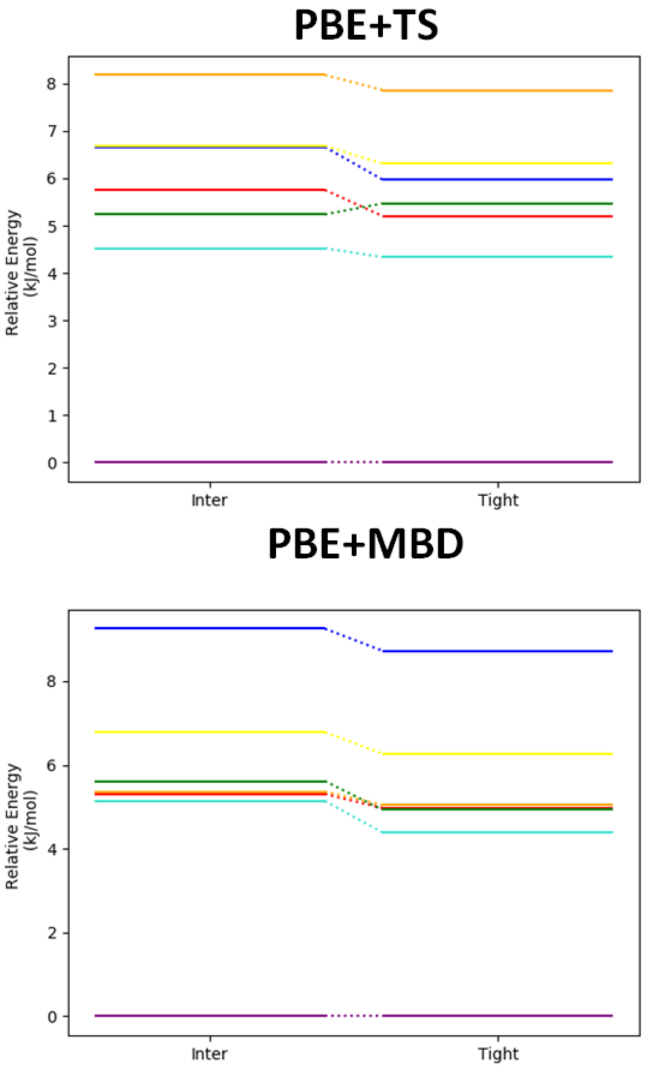

Figure S1: Benchmark of intermediate settings (inter) and *higher-level* (tight) settings in FHI-aims for representative structures fo α-HMX.

2 Interaction Chain Analysis

We have previously developed a method to extract and calculate the energies of intermolecular interactions in molecular crystals.[8] This can be used to elucidate the contributions of specific intermolecular interactions and the differences in their treatment by different dispersion methods and DFT functionals.[8,9] Nearest neighbor interactions in the crystal structure are used to construct periodic molecular chains. These periodic interaction chains better reflect the periodicity of intermolecular interactions in crystals, compared to calculations of interactions between isolated pairs of molecules. A unit cell is constructed around each chain such that the lattice vector in the direction(s) of the interaction are the same as in the bulk crystal while a vacuum of 40 Åis added in the other lattice vector direction(s). This isolates the specific intermolecular interactions. The k-point grid was defined such that in the direction(s) of the interactions the number of k-points was the nearest integer to 25 divided by the lattice vector length(s). One k-point was used in the direction(s) of the vacuum. Single point energy calculations with PBE+TS, PBE+MBD, and PBE0+MBD were performed to determine the interaction energy, IE, as follows:

$$IE = E_{\text{Periodic Chain}} - Z \times E_{\text{Molecule}} \tag{1}$$

where $E_{PeriodicChain}$ is the total energy of the extracted periodic chain, Z is the number of molecules in the constructed unit cell, and $E_{Molecule}$ is the total energy of an isolated molecule extracted from the crystal structure, which accounts for intramolecular interactions.[8]

3 LLM-105

3.1 Genarris

A single Genarris run was conducted for this target with an s_r of 0.85 and a target volume of 774, obtained with PyMoVE, a machine learned volume estimation model written in python.[10] The experimental volume for this target is 748, which means that PyMoVE had an error of 3.43%. In Figure S2 the space group, volume, and lattice parameter distribution are shown for each stage of the Robust workflow of Genarris.[11] All structures are optimized with the generalized gradient approximation (GGA) of Perdew, Burke, and Ernzerhof (PBE)[3] paired with the pairwise diserpsion correction of Tkatchenko and Scheffler (TS)[4] (PBE+TS).

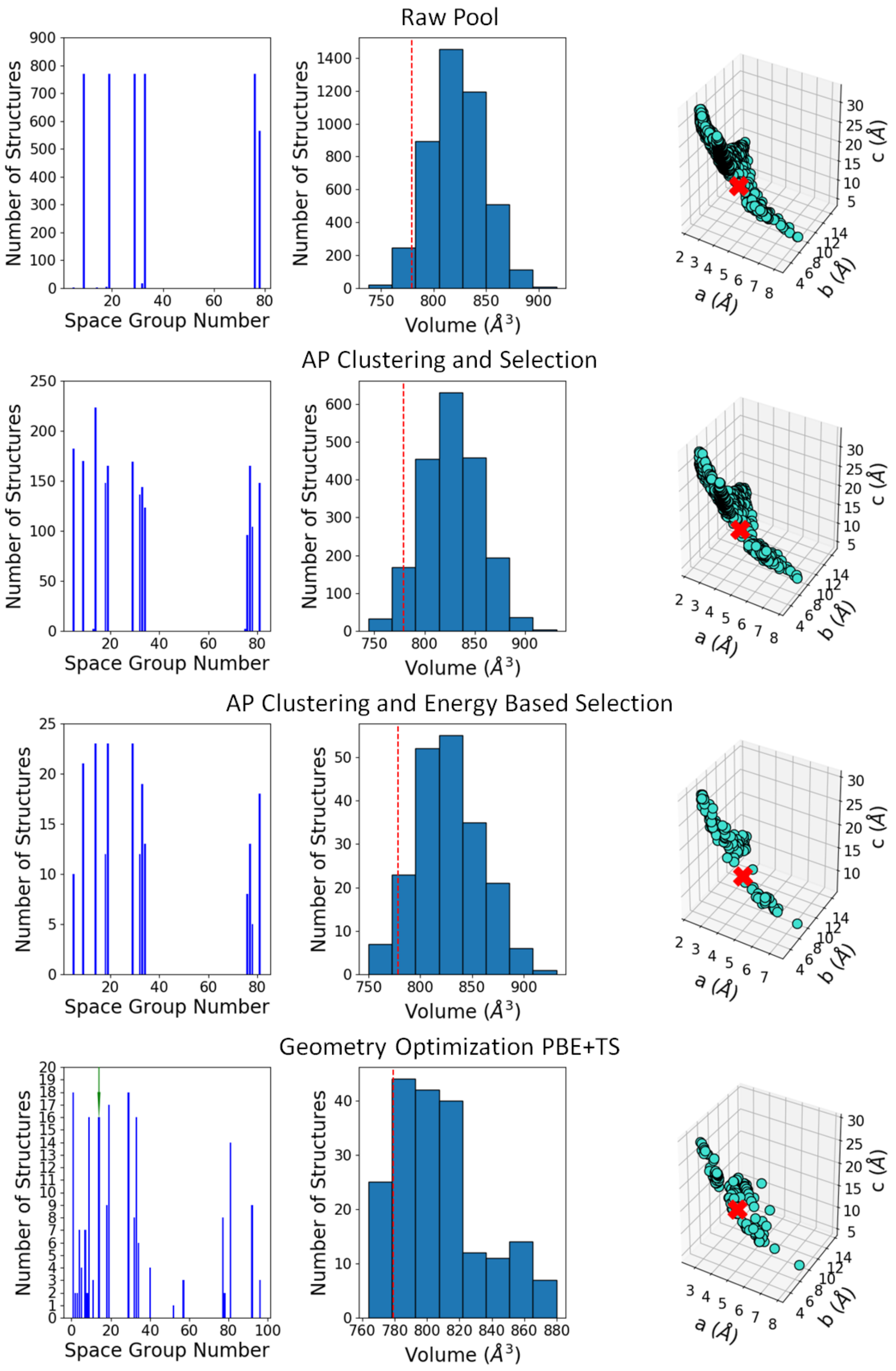

Figure S2: Space group (left), volume (right), and lattice parameter distribution (right) of the Robust workflow of Genarris for LLM-105. Special positions are indicated with orange, general positions with blue, and the experimental space group is indicated with a green arrow. The experimental volume is indicated with a dashed red line. The experimental structure, which was generated by Genarris, is shown with a red cross on the lattice parameter distribution.

3.2 RMSD

Table S1 shows the RMSD values comparing the experimental structure as generated by GAtor to the experimental structure extracted from the Cambridge Structural Database (CSD) for LLM-105. As can be seen, the run with symmetric crossover

produced the lowest RMSD. Table S2 shows the RMSD values when the experimental structure as generated by GAtor is relaxed with different DFT functionals and dispersion methods. As can be seen, PBE+TS and PBE+MBD produced the lowest values of RMSD.

GA Run	RMSD (Å)
Mutation	0.011
Standard	N/A
Symmetric	0.004

Table S1: RMSD values for GAtor generated structures compared to the experimental structure for LLM-105.

DFT Level of Theory	RMSD (Å)
PBE+TS	TS: 0.014
PBE+MBD	MBD: 0.014
PBE+XDM	XDM: 0.024

Table S2: RMSD values for GAtor generated structures compared to the experimental structure for LLM-105 with different DFT functionals and dispersion methods.

3.3 High Resolution Lattice Parameter Distribution Plots

Due to spacing constraints, lattice parameter distribution plots are made to column width. However, we present higher resolution and full width plots below, in Figure S3. Similar to those showed in the manuscript, the lattice parameter distributions are color coded according to relative energy, with darker colors corresponding to lower energies and lighter colors corresponding to higher energies. If generated, the experimental structure is indicated with a green cross.

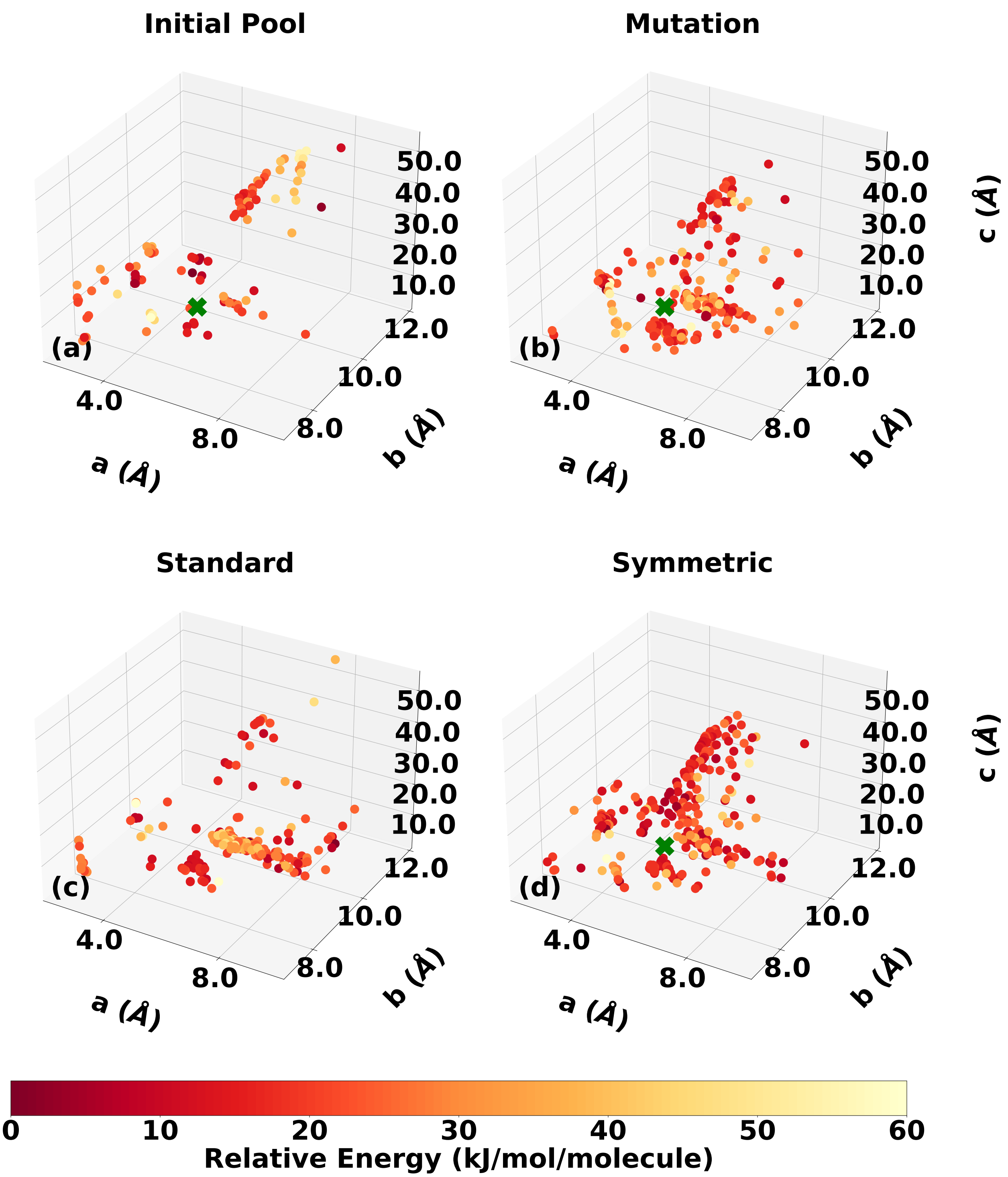

Figure S3: High resolution lattice parameter distribution plots for LLM-105.

3.4 Full Vibrational Energy Ranking

Because we have data up to 500 K for the vibrational free energy we present relative energy rankings (PBE0+MBD+$E_{vib}(T)$) across this temperature range, as seen in Figure S4. The colors correspond to those shown in the manuscript. The experimental structure, shown in green, is the most stable at every temperature. The orange and purple structures are significantly stabilized at higher temperatures while the blue structure is significantly destabilized. The red structure remains fairly stable up to 500 K.

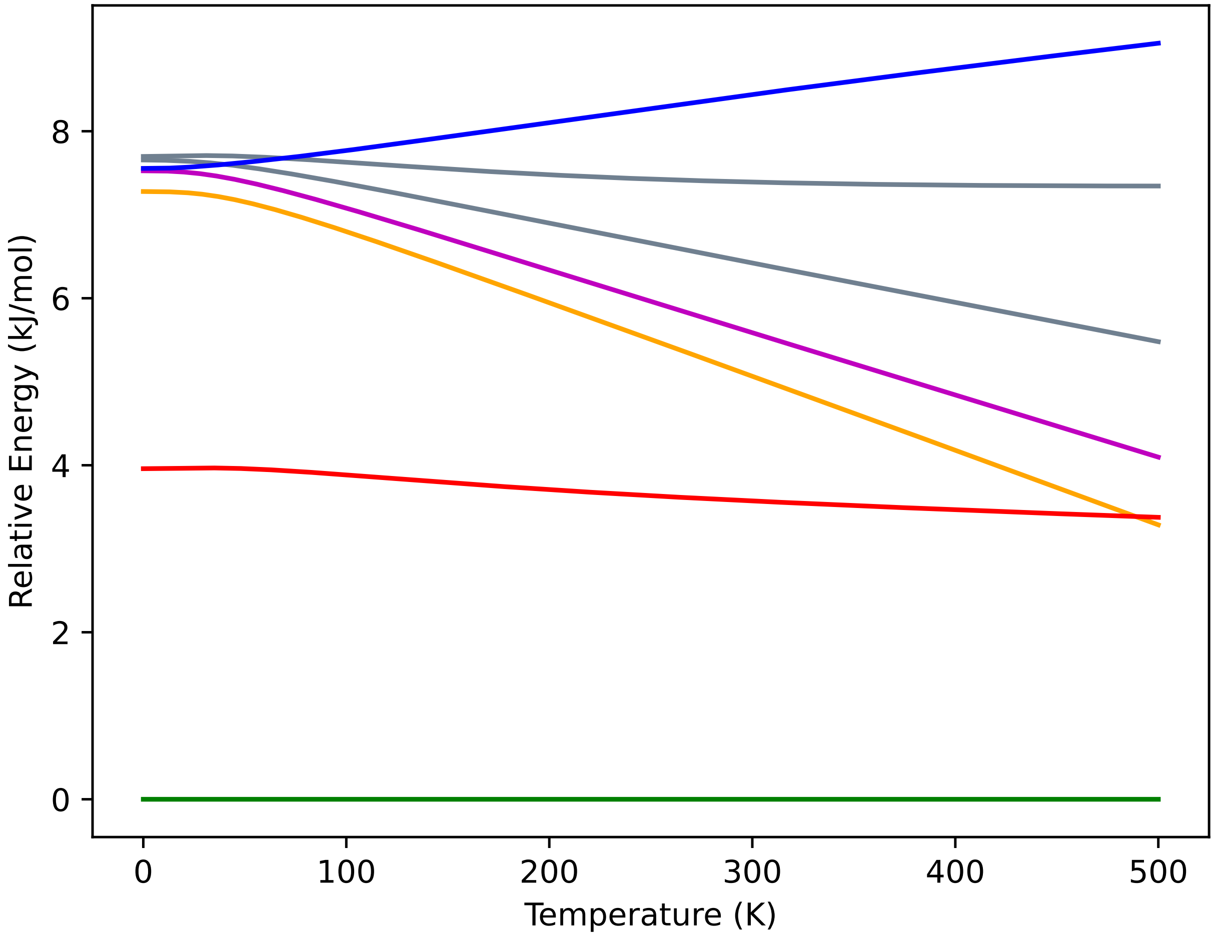

Figure S4: Caption

3.5 Full Energy vs Density Plots

Density is an important property of EMs. Figure S5 shows the density of GAtor generated structures as a function of PBE+TS energy for LLM-105. The experimental structure, if generated, is indicated with a green cross with other structures colored according to their relative energy. As can be seen, density does appear to be correlated with energy for LLM-105. The experimental structure is among the most dense and is always the lowest in energy.

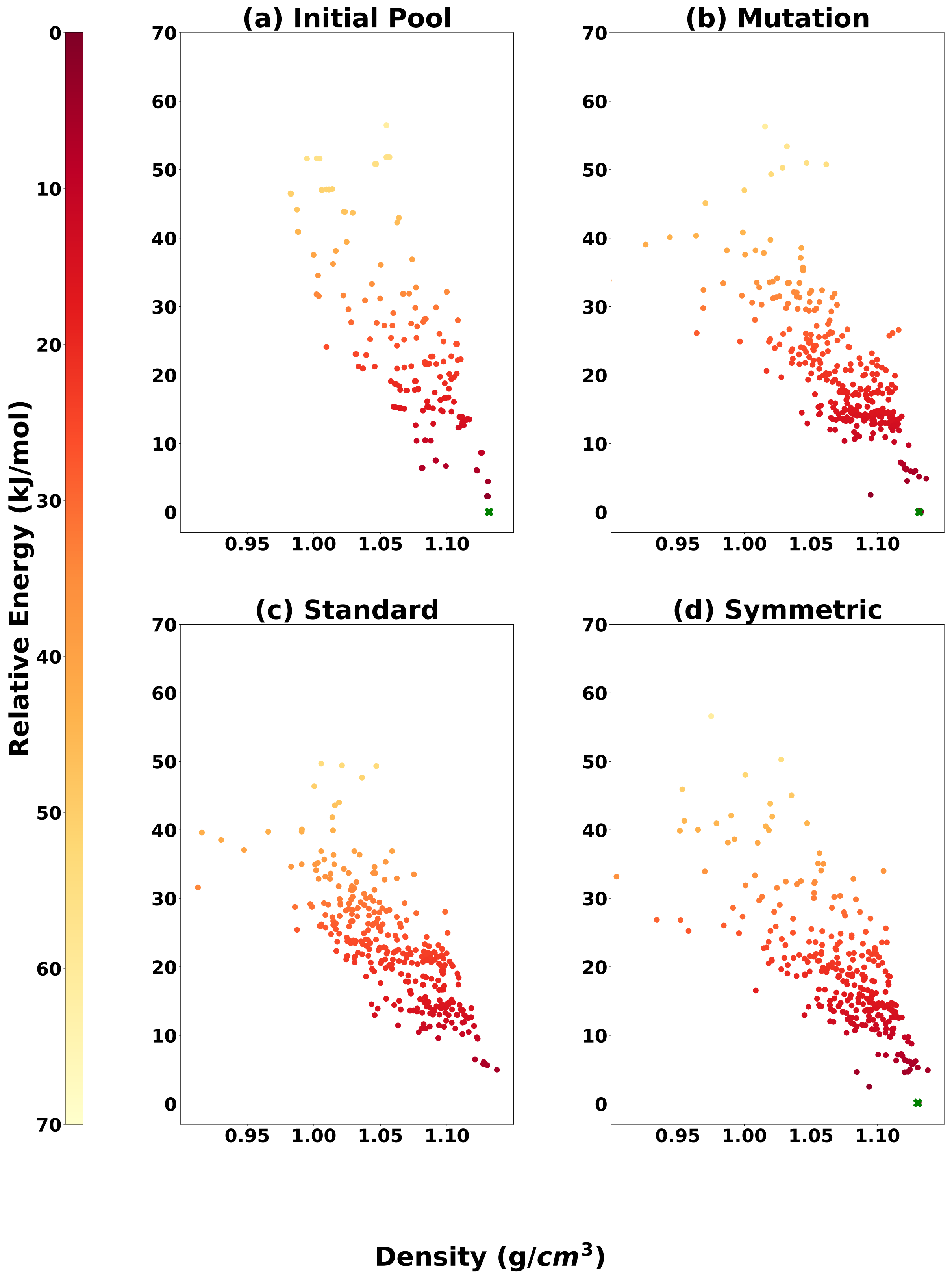

Figure S5: Density of structures generated by GAtor as a function of relative energy for (a) the initial pool, (b) mutation, (c) standard crossover, and (d) symmetric crossover for LLM-105. Structures are colored according to their relative energy with darker colors corresponding to lower energies. The experimental structure, if generated, is indicated by a green cross.

3.6 Interaction Energy

LLM-105 is dominated by nitro-nitro, nitro-amine, and π-π stacking interactions. Previously, we ave found that both nitro-nitro and π-π stacking interactions are stabilized with PBE+TS and destabilized with PBE+MBD compared to PBE0+MBD, whereas nitro-amine interactions are destabilized with both PBE+TS and PBE+MBD.[9] As seen in Figure 6 the structure shown in purple is destabilized relative to other structures when switching from PBE+TS to PBE+MBD. This could be attributed to the destabilization of the nitro-amine interactions in the purple structure when switching from PBE+TS to PBE+MBD, as shown in Figure S6. In contrast, these interactions are slightly stabilized in the blue structure. The experimental structure does not have nitro-amine interactions but rather nitro-nitro interactions, which are stabilized when switching from PBE+MBD to PBE0+MBD. This may explain the destabilization of other structures compared to the experimental structure seen in Figure 6. The structure shown in orange has both nitro-nitro and nitro-amine interactions. Similar to the experimental structure, the interactions in this structure are stabilized when switching from PBE+MBD to PBE0+MBD, though to a lesser extent than the experimental structure.

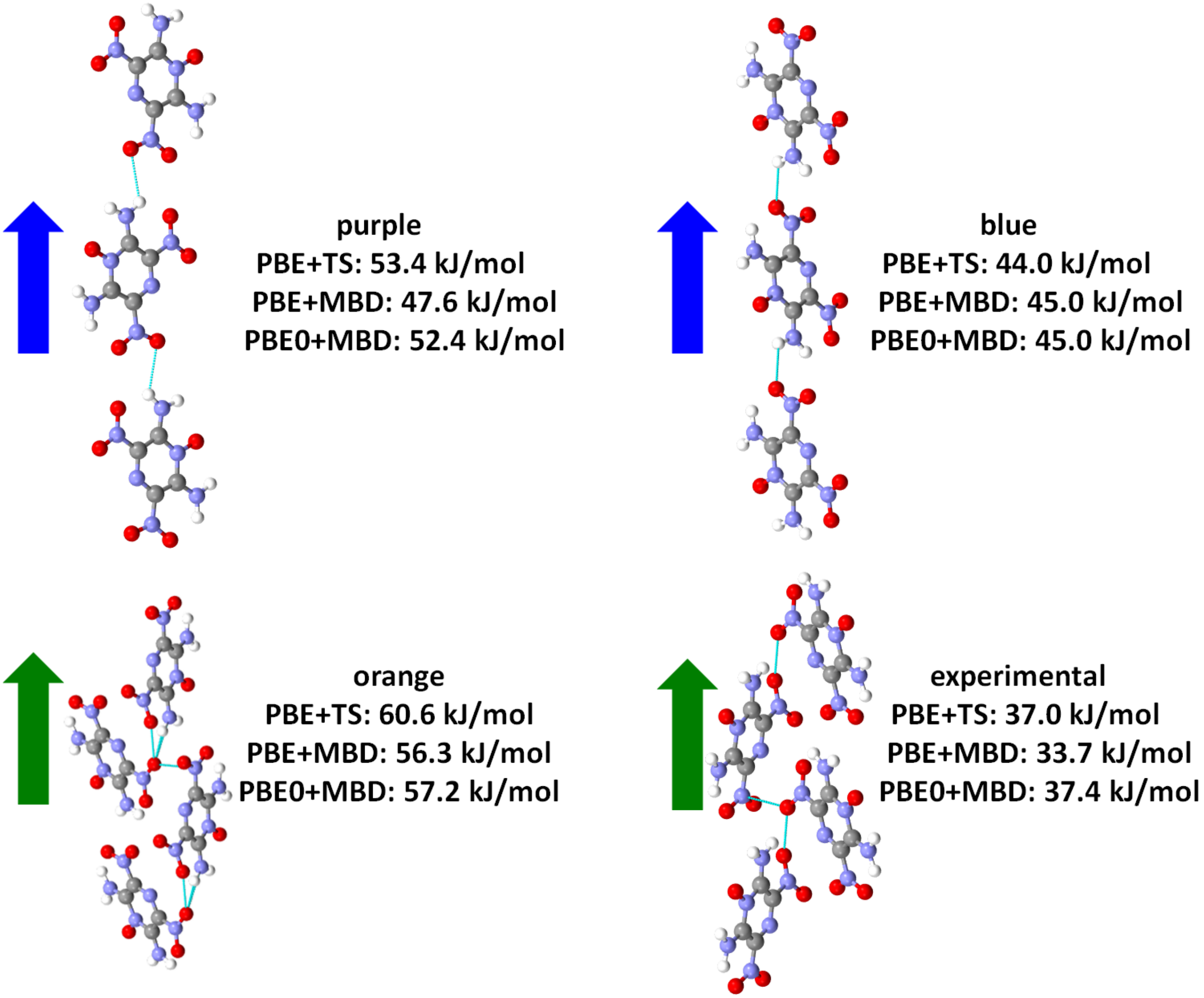

Figure S6: Interaction energies of nitro-amine and nitro-nitro interactions in the structures colored in purple, and blue as well as the experimental structure of LLM-105. The b and c lattice vectors are shown by green and blue arrows, respectively.

4 α-RDX

4.1 Genarris

For this target a single Genarris run was conducted for this target with an s_r of 0.85 and a target volume of 1600 $\AA^3$. The volume obtained with PyMoVE was 1193 $\AA^3$ which was an error of 25.3%. In Figure S7 the space group, volume distribution, and lattice parameter distribution are shown for each stage of the Robust workflow of Genarris.[11]

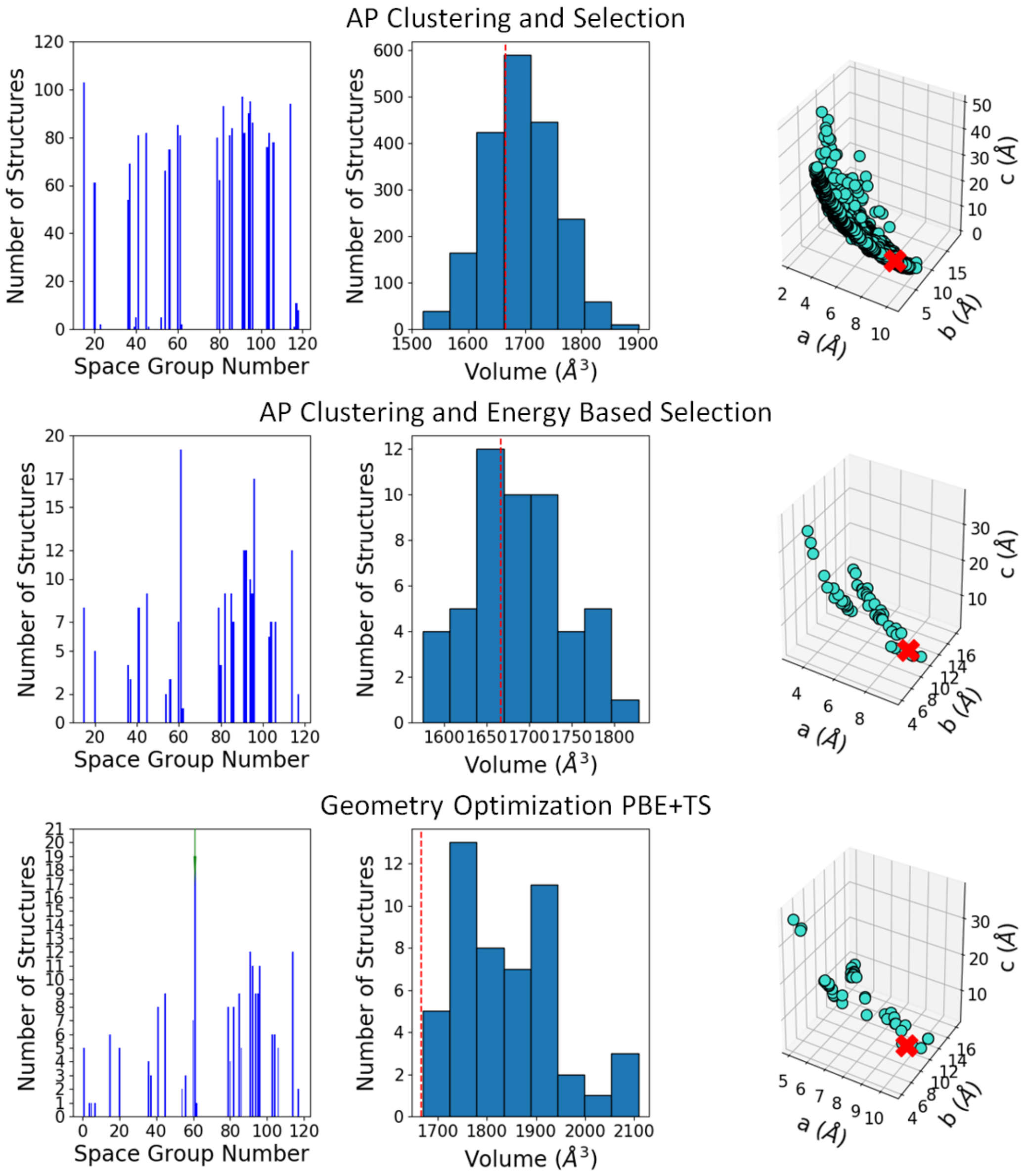

Figure S7: Space group (left), volume distribution (center), and lattice parameter distribution (right) of the Robust workflow of Genarris for α-RDX. Special positions are indicated with orange, general positions with blue, and the experimental space group is indicated with a green arrow. The experimental volume is indicated with a dashed red line. The experimental structure, which was generated by Genarris, is shown with a red cross on the lattice parameter distribution.

4.2 RMSD

Table S3 shows the RMSD values comparing the experimental structure as generated by GAtor to the experimental structure extracted from the Cambridge Structural Database (CSD) for α-RDX. As can be seen, the run with mutation produced the lowest RMSD. Table S4 hows the RMSD values when the experimental structure as generated by GAtor is relaxed with different DFT functionals and dispersion methods. As can be seen, PBE+MBD produced the lowest RMSD.

GA Run	RMSD (Å)
Mutation	0.005
Standard	N/A
Symmetric	0.025

Table S3: RMSD values for GAtor generated structures compared to the experimental structure for α-RDX.

DFT Level of Theory	RMSD (Å)
PBE+TS	TS: 0.042
PBE+MBD	MBD: 0.026
PBE+XDM	XDM: 0.034

Table S4: RMSD values for GAtor generated structures compared to the experimental structure for α-RDX with different DFT functionals and dispersion methods.

4.3 High Resolution Lattice Parameter Distribution Plots

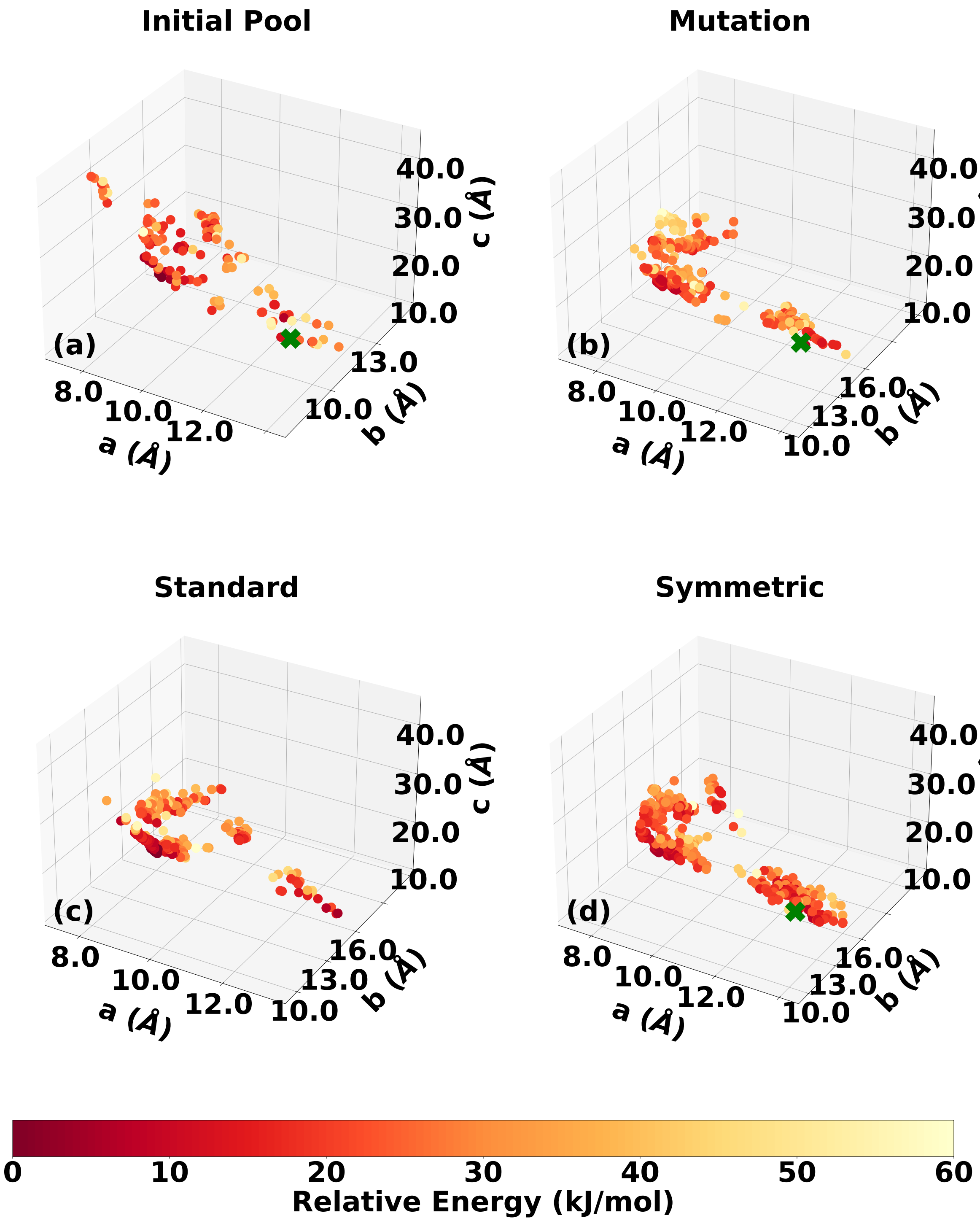

Figure S8: High resolution lattice parameter distribution plots for α-RDX.

4.4 Full Energy vs Density Plots

Figure S9 shows the density of GAtor generated structures as a function of PBE+TS energy for α-RDX. The experimental structure, if generated, is indicated with a green cross with other structures colored according to their relative energy. As can be seen, density does appear to be correlated with energy for α-RDX. The experimental structure is among the most dense and is always the lowest in energy. However, there are several structures close in energy to the experimental with higher densities.

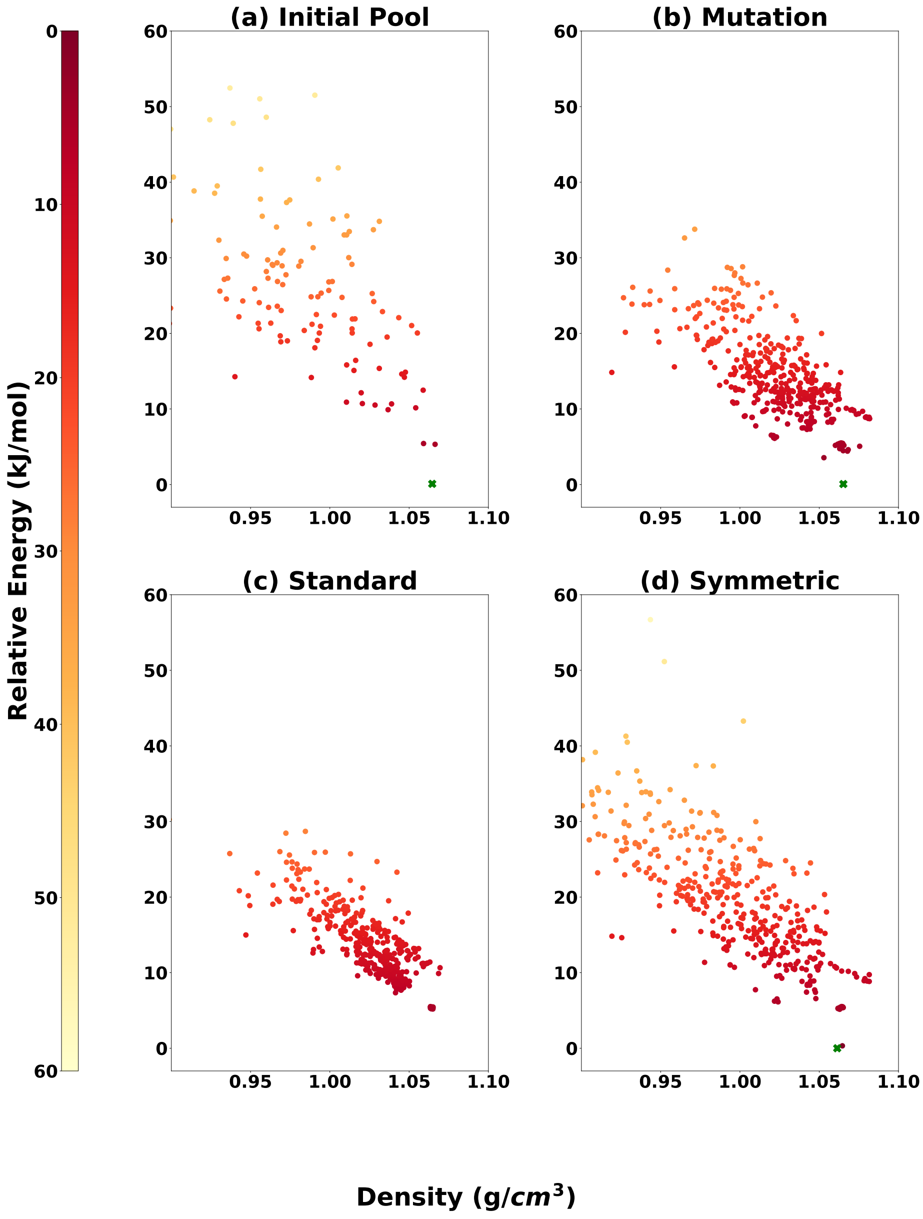

Figure S9: Density of structures generated by GAtor as a function of relative energy for (a) the initial pool, (b) mutation, (c) standard crossover, and (d) symmetric crossover for α-RDX. Structures are colored according to their relative energy with darker colors corresponding to lower energies. The experimental structure, if generated, is indicated by a green cross.

4.5 Interaction Energy

Figure S10 shows the interaction energies along the three lattice vectors in the structure shown in blue in Figure 11, compared to the experimental structure. The interaction energies shown are the absolute values of those calculated so that more positive values indicate higher stability. The two structures have similar intermolecular interactions and packing motifs. In both structures, nitro-hydrogen interactions are dominant. The interactions in the experimental structure are overall less sensitive

to the choice of dispersion method and DFT functional than those in the structure colored in blue. The most dramatic change in the interaction energies is found for the nitro-hydrogen interaction along the c lattice vector of the structure colored in blue in Figure 11. This interaction is destabilized by 17 kJ/mol when switching from the TS dispersion method to the MBD method. This could explain the significant change in the ranking of this structure in Figure 11.

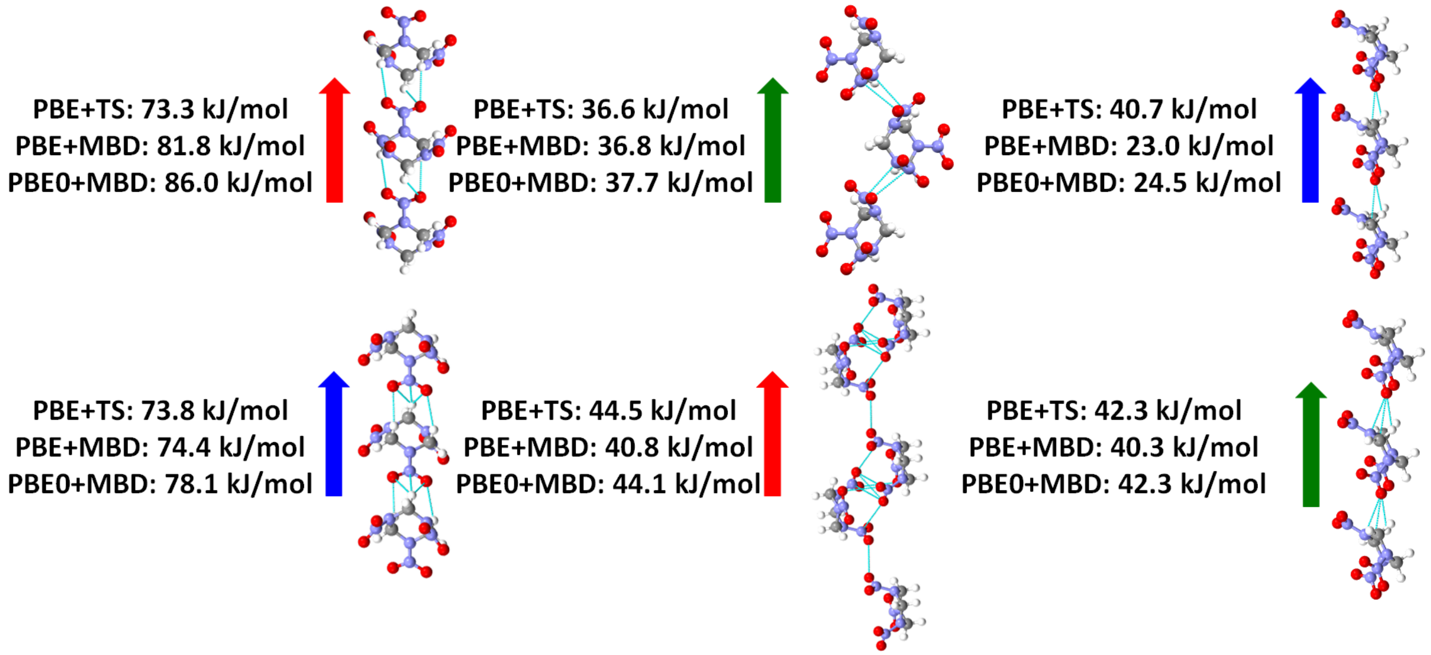

Figure S10: Interaction energies of nitro-nitro and nitro-hydrogen interactions in the structure colored in blue in Figure **??** (top) and the experimental structure (bottom) of α-RDX. The a, b, and c lattice vectors are indicated by red, green, and blue arrows, respectively.

5 α-HMX

5.1 Genarris

A single Genarris run was conducted for this target with an s_r of 0.85 and a target volume of 2132 $\AA^3$, obtained with PyMoVE.[10] The experimental volume for this target is 2138, which means that PyMoVE had an error of 0.29%. In Figure S11 the space group, volume, and lattice parameter distributions are shown for each stage of the Robust workflow of Genarris.[11]

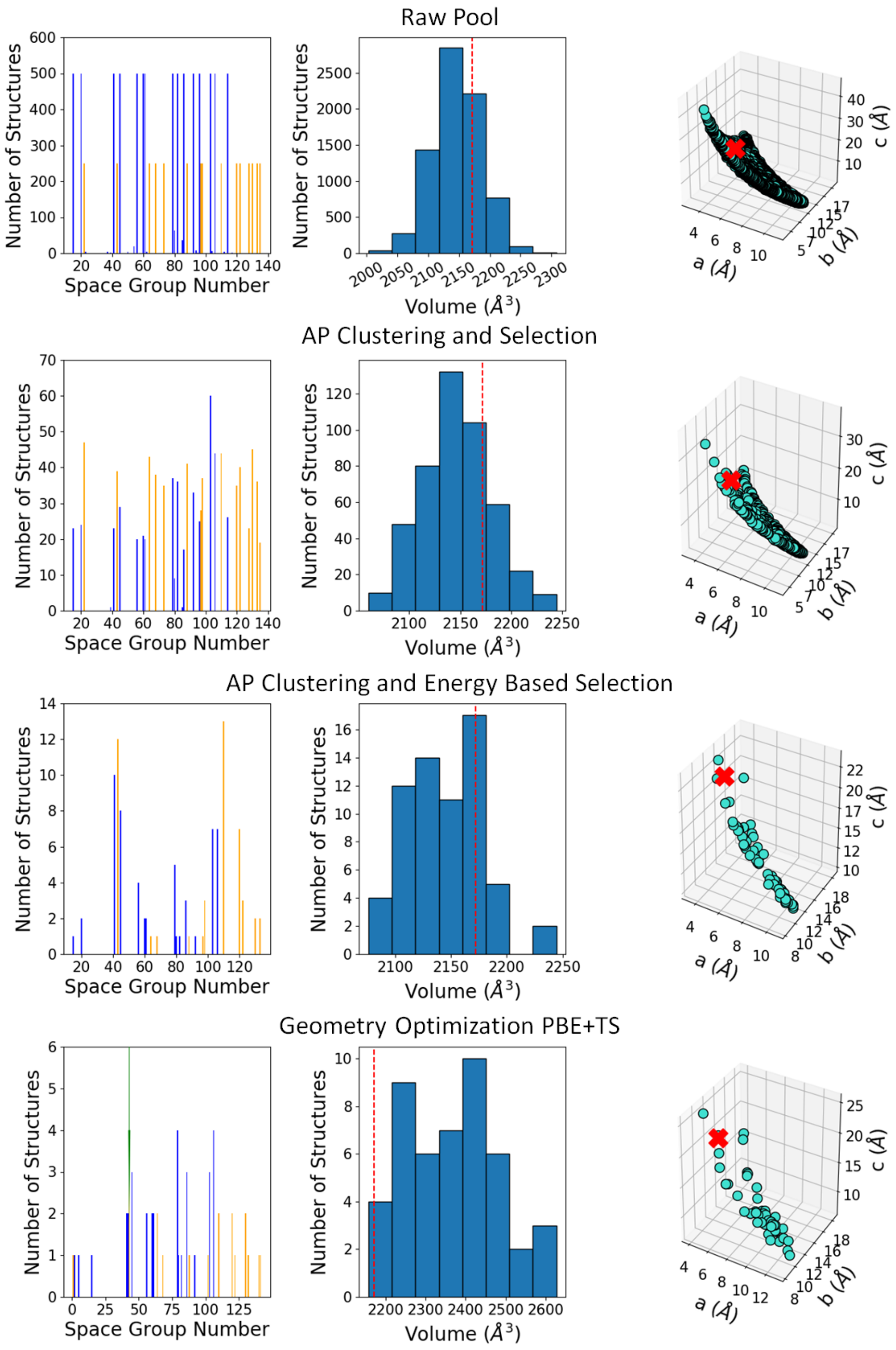

Figure S11: Space group (left), volume (center), and lattice parameter distribution (right) for each stage of the Genarris workflow for α-HMX. Special positions are shown in orange, general positions are shown in blue, and the experimental space group is indicated with a green arrow. The experimental volume is indicated with a dashed red line. The experimental structure, which was generated by Genarris, is shown with a red cross.

5.2 RMSD

Table S5 shows the RMSD values comparing the experimental structure as generated by GAtor to the experimental structure extracted from the Cambridge Structural Database (CSD) for α-HMX. As can be seen, the run with symmetric crossover produced the lowest RMSD. Table S6 hows the RMSD values when the experimental structure as generated by GAtor is

relaxed with different DFT functionals and dispersion methods. As can be seen, PBE+TS produced the lowest value of RMSD.

GA Run	RMSD (Å)
Mutation	N/A
Standard	0.027
Symmetric	0.025

Table S5: RMSD values for GAtor generated structures compared to the experimental structure for α-HMX.

DFT Level of Theory	RMSD (A)
PBE+TS	TS: 0.019
PBE+MBD	MBD: 0.029
PBE+XDM	XDM: 0.045

Table S6: RMSD values for GAtor generated structures compared to the experimental structure for α-HMX with different DFT functionals and dispersion methods.

5.3 High Resolution Lattice Parameter Plots

High resolution, large-scale lattice parameter distribution plots for structures generated by GAtor for α-HMX. Darker colors correspond to lower eneriges and lighter colors correspond to higher energies. If generated, the experimental structure is indicated with a green cross.

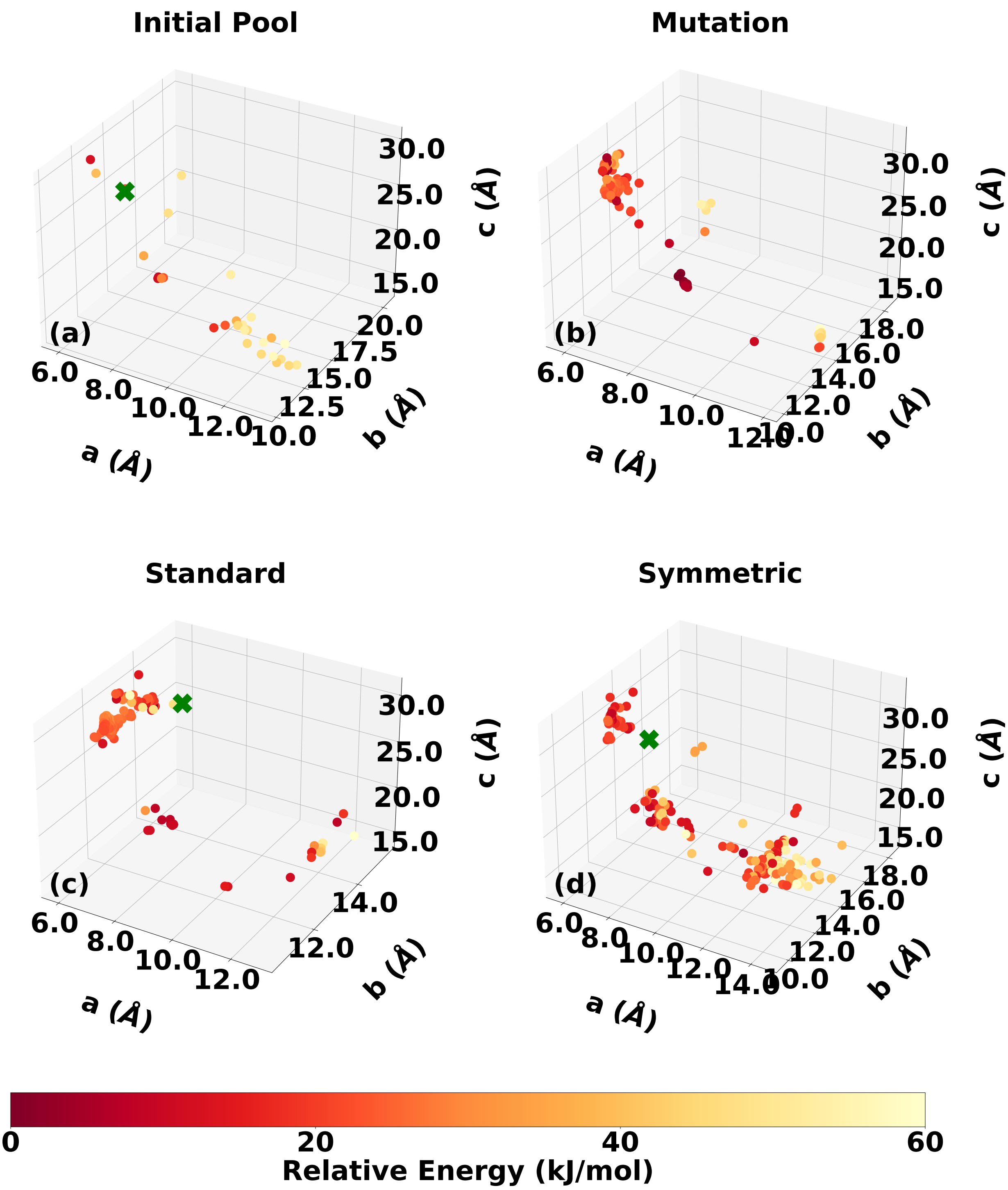

Figure S12: High resolution lattice parameter distributions plots at full-scale for structures of α-HMX.

5.4 Full Vibrational Energy Ranking

Figure S13 shows the PBE0+MBD+$E_{vib}(T)$ rankings up to 500 K for α and β HMX. The colors correspond to those shown in the manuscript. The experimental structure of the α form, shown in purple, is the most stable at almost every temperature. When the temperature approaches 500 K, the orange structure becomes the most stable. The pink and blue structures are stabilized as temperature increases while the experimental structure of the β form and the structure shown in green are destabilized. The β form is expected to destabilize at higher temperatures since HMX has two high temperature polymorphs, α and δ.

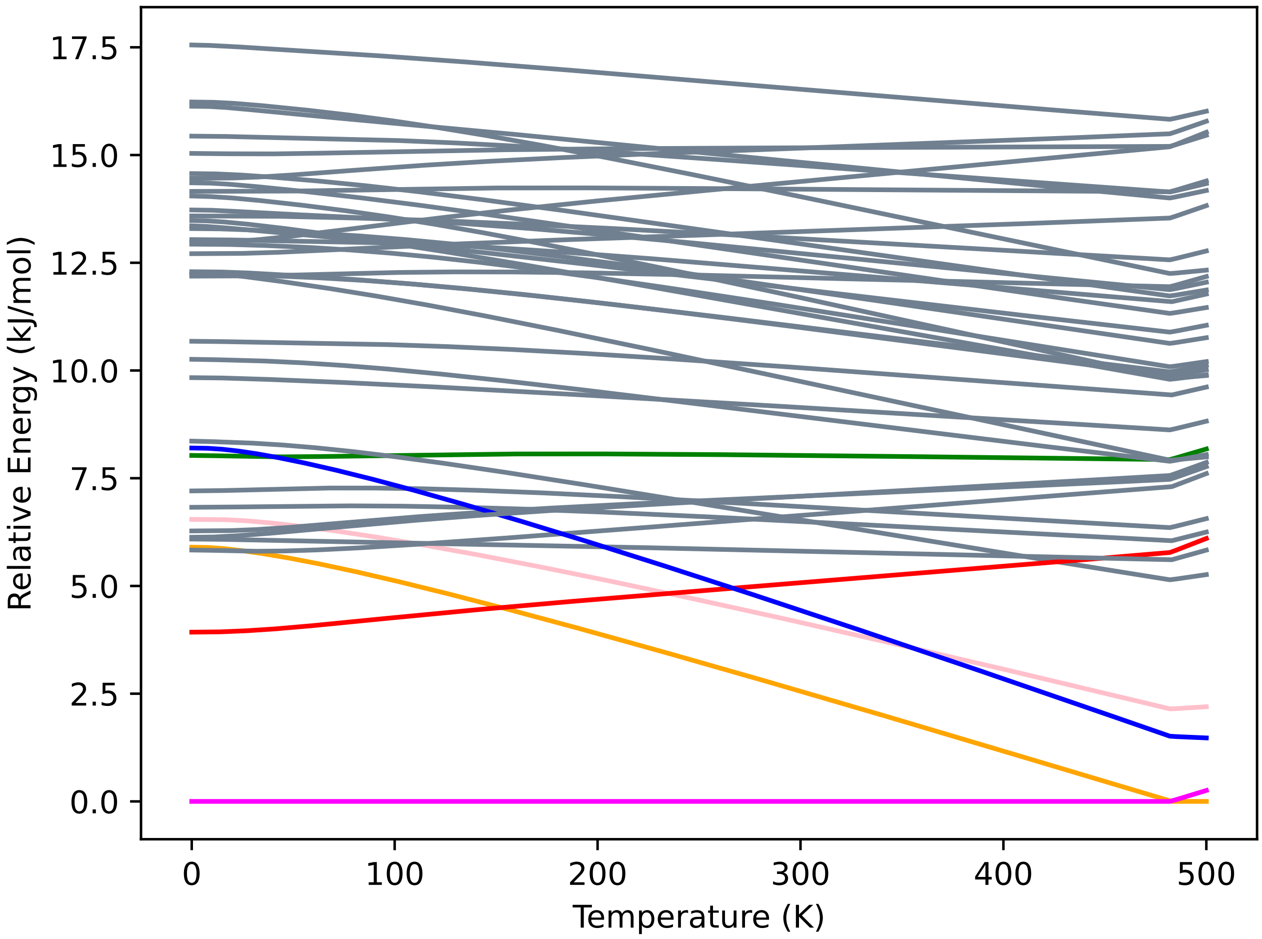

Figure S13: Caption

5.5 Full Energy vs Density Plots

Figure S14 shows the density of GAtor generated structures as a function of PBE+TS energy for α-HMX. The experimental structure, if generated, is indicated with a green cross with other structures colored according to their relative energy. As can be seen, density does appear to be correlated with energy for α-HMX but to a lesser extent than LLM-105 or α-RDX. Both the standard and symmetric crossover runs appear to generate denser, relatively low energy structures than the experimental.

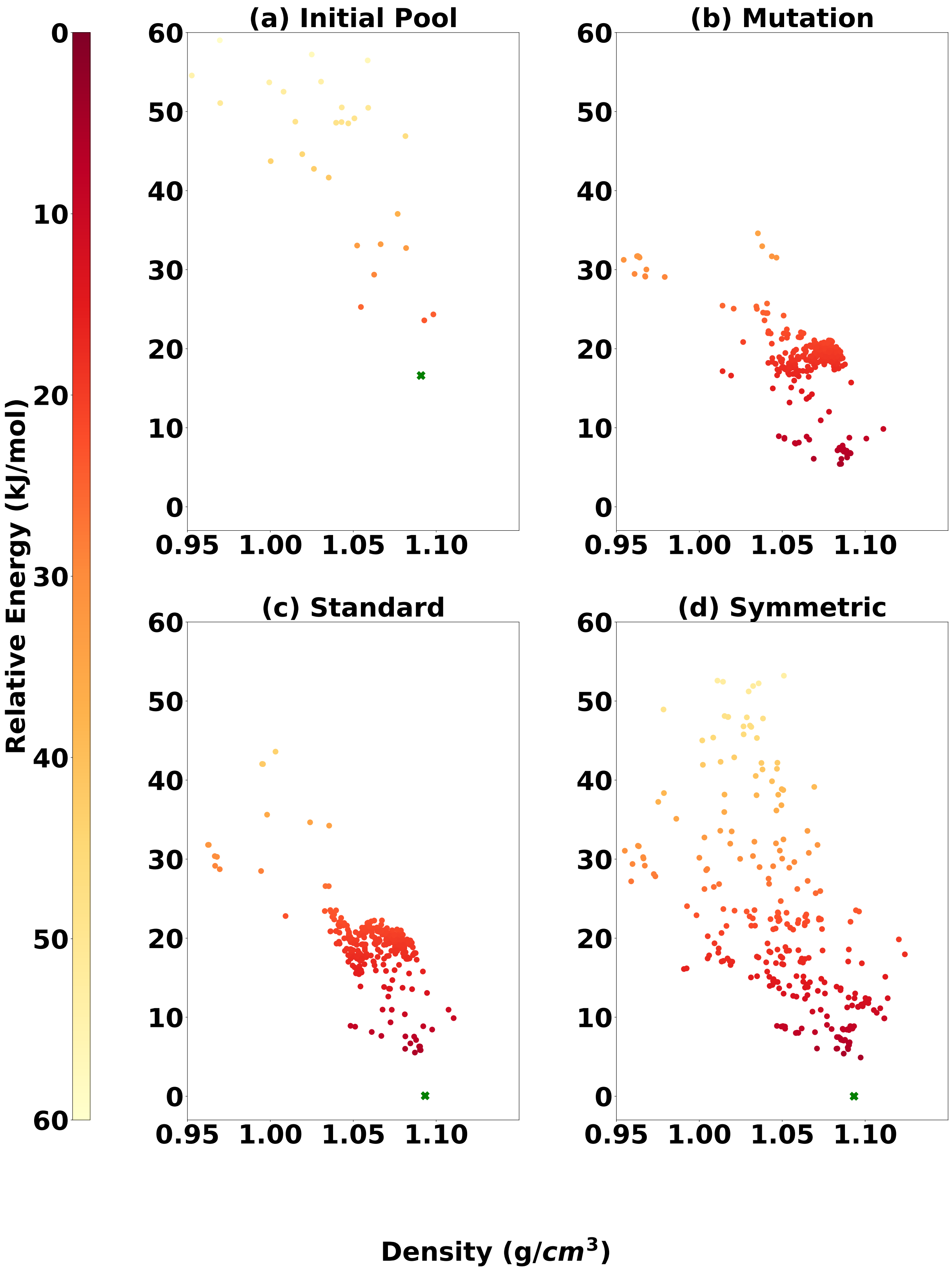

Figure S14: Density of structures generated by GAtor as a function of relative energy for (a) the initial pool, (b) mutation, (c) standard crossover, and (d) symmetric crossover for α-HMX. Structures are colored according to their relative energy with darker colors corresponding to lower energies. The experimental structure, if generated, is indicated by a green cross.

5.6 Interaction Energy

Similar to α-RDX, α-HMX is dominated by nitro-nitro and nitro-hydrogen interactions. Previously, we have found that nitro-nitro interactions are stabilized with PBE+TS but destabilized with PBE+MBD while nitro-hydrogen interactions are destabilized with both PBE+TS and PBE+MBD.[9] As seen in Figure 16 the blue and purple structures have similar packing motifs but are ranked differently with PBE+MBD compared to PBE0+MBD. Because the packing motifs are so similar, the intermolecular interactions in these structures, illustrated in Figure S15, are almost identical. The most dominant interaction, nitro-hydrogen interaction 2D chains along the a and b lattice vectors, is ranked similarly by all three methods. As expected, PBE+TS and PBE+MBD destabilize these interactions in comparison to PBE0+MBD.[9] The purple structure is slightly more destabilized when switching from PBE+TS to PBE+MBD, which could partly explain the trend seen in Figure 16. Furthermore, the packing motif of the purple structure allows for some nitro-nitro interactions to take place along the b lattice vector. As seen in Figure S15, this interaction is destabilized with PBE+MBD. This could partly explain why the purple structure is destabilized when switching from PBE+TS to PBE+MBD in comparison to the blue structure.

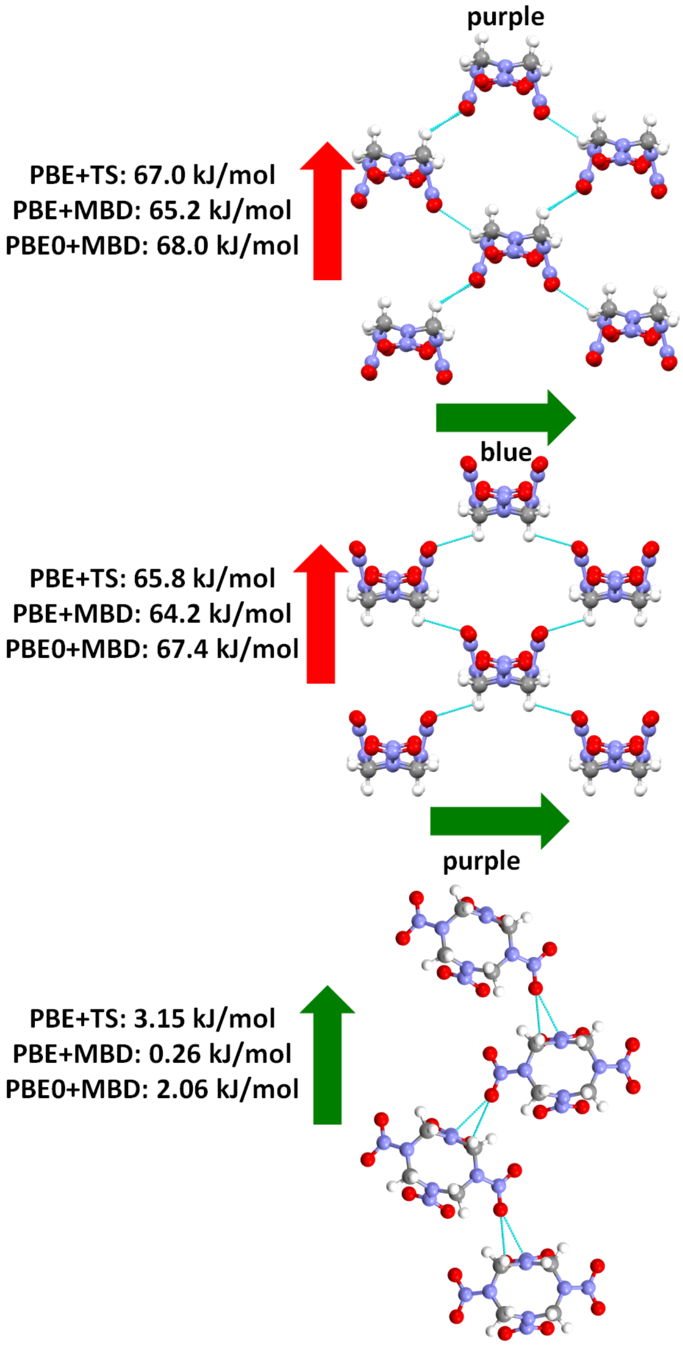

Figure S15: Nitro-nitro and nitro-hydrogen interactions in the structures shown in purple and red in Figure **??**. The a and b lattice vectors are shown in red and green, respectively.

6 β-HMX

One of the constraints of our approach is that only one conformer can be reasonably used for structure generation. However, future iterations of Genarris and GAtor will allow for conformational flexibility. To verify that Genarris and GAtor can find conformational polymorphs, we performed a single Genarris and GAtor run using the conformer of β-HMX. β-HMX is chosen due to its small system size ($Z = 2$) as well as the fact that there is only one molecule in the asymmetric unit. Future iterations of Genarris and GAtor will also be able to handle multiple molecules in the asymmetric unit. The same settings were used for the Genarris calculation of β-HMX as detailed in the manuscript. The PyMoVE volume estimation was 532 $\AA^3$ while the experimental value was 519 $\AA^3$ for a percent error of 2.5%. For the sake of computational expense, only one GAtor run was conducted for this target using the energy-based fitness function with 100% chance of mutation.

6.1 Genarris

Genarris was able to generate the experimental structure for β-HMX, as seen below in Figure S16 where each stage of the Robust workflow is shown.[11] The final pool, which was optimized with PBE+TS, does not have a lot of density near the portion of the potential energy surface where the experimental structure resides. Additionally, all of the structures in the final pool have a higher volume than the experimental structure. However, there is good coverage of the experimental space group.

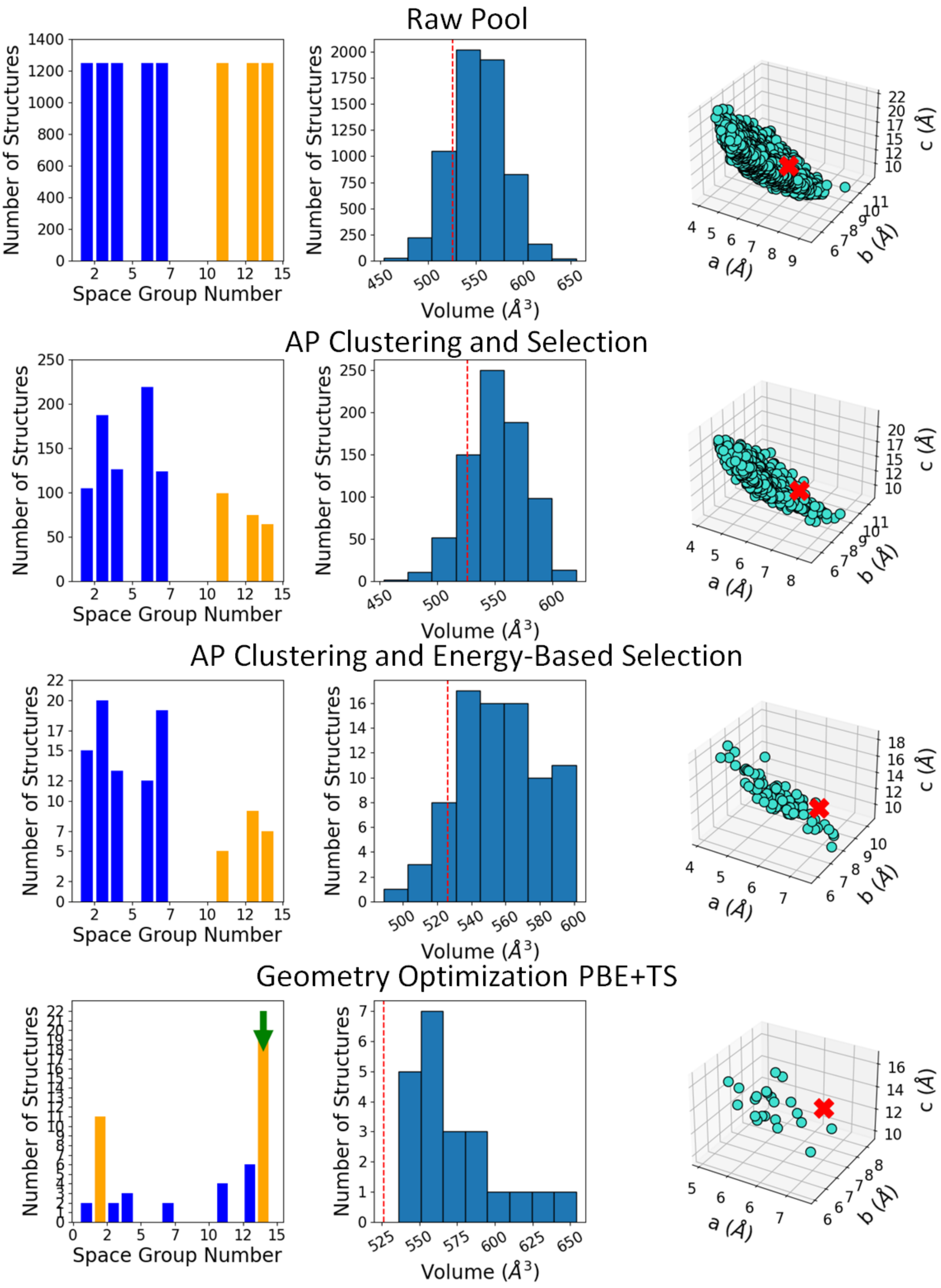

Figure S16: Space group (left), volume (center), and lattice parameter distribution (right) for each stage of the Genarris workflow for β-HMX. Special positions are shown in orange, general positions are shown in blue, and the experimental space group is indicated with a green arrow. The experimental volume is indicated with a dashed red line. The experimental structure, which was generated by Genarris, is shown with a red cross.

6.2 RMSD

Table S7 shows the RMSD values comparing the experimental structure as generated by GAtor to the experimental structure extracted from the Cambridge Structural Database (CSD) for β-HMX. As can be seen, the run with standard crossover produced the lowest RMSD. In general, the RMSD values for this target are much lower than those of the other targets.

GA Run	RMSD (Å)
Mutation	0.006
Standard	0.004
Symmetric	0.005

Table S7: RMSD values for GAtor generated structures compared to the experimental structure for β-HMX.

DFT Level of Theory	RMSD (Å)
PBE+TS	0.011
PBE+MBD	0.017
PBE0+MBD	0.023

Table S8: RMSD values for GAtor generated structures to the experimental structure for β-HMX with different DFT functionals and dispersion methods.

6.3 High Resolution Lattice Parameter Plots

High resolution, large-scale lattice parameter distribution plots for structures generated by GAtor for β-HMX. Darker colors correspond to lower energies and lighter colors correspond to higher energies. If generated, the experimental structure is indicated with a green cross.

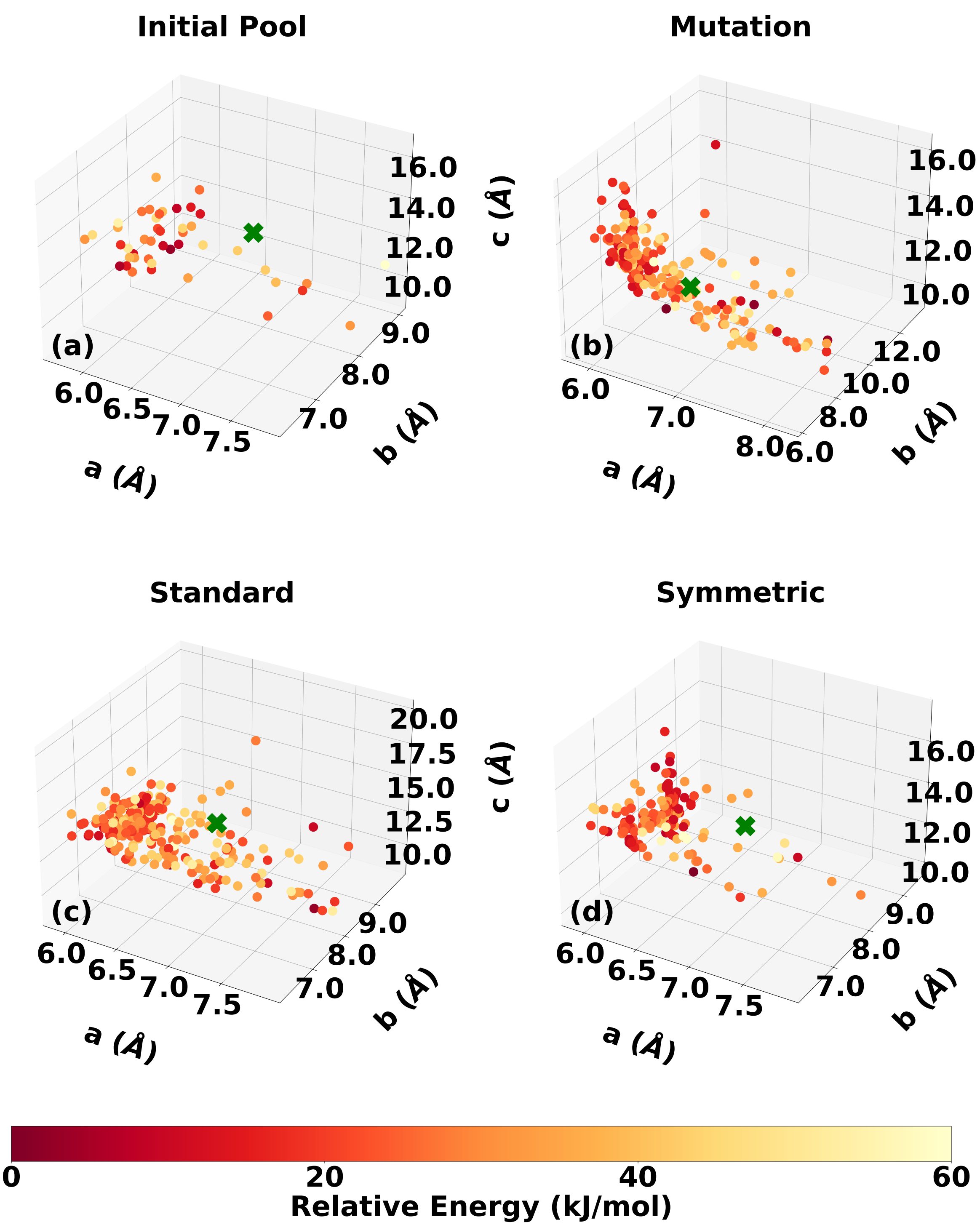

Figure S17: High resolution lattice parameter distributions plots at full-scale for structures of β-HMX.

6.4 Full Energy vs Density Plots

Figure S18 shows the density of GAtor generated structures as a function of PBE+TS energy for β-HMX. The experimental structure, if generated, is indicated with a green cross with other structures colored according to their relative energy. As can be seen, density does appear to be correlated with energy for β-HMX, or at least to greater extent than in α-HMX. All three GA runs generated similar amounts of high density structures, with the experimental structure being among the most dense. In the initial pool the experimental structure appears to be both the lowest in energy and the highest in density.

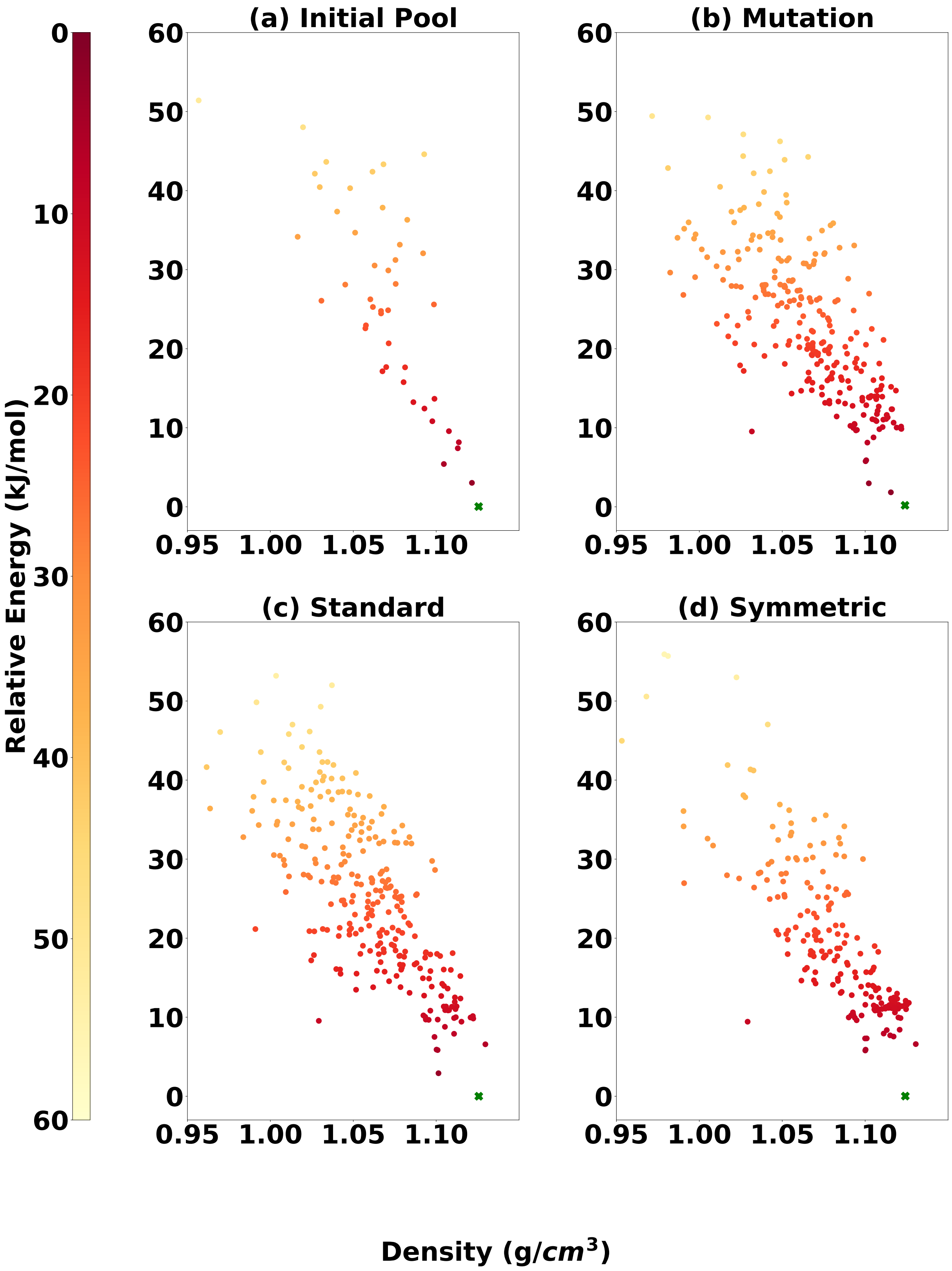

Figure S18: Density of structures generated by GAtor as a function of relative energy for (a) the initial pool, (b) mutation, (c) standard crossover, and (d) symmetric crossover for β-HMX. Structures are colored according to their relative energy with darker colors corresponding to lower energies. The experimental structure, if generated, is indicated by a green cross.

7 HMX

7.1 Quasi-Harmonic Approximation

As discussed in the main text, the α polymorph was ranked as more stable than the β polymorph, which disagrees with experimental observations. To further investigate this, we calculated the Gibbs free energy at 295 K in the solid-state for the two experimental forms of HMX. The Gibbs free energy is given by Equation 2:

$$G_{295} = -E_{DFT}/Z - E_{vib}(T) - \int_0^T C_p dT \tag{2}$$

where G_{295} is the Gibbs free energy at 295 K, E_{DFT} is the total energy of the DFT, Z is the number of molecules in the unit cell, $E_{vib}(T)$ is the vibrational energy at 295 K, and $\int_0^T C_p dT$ is the integral from 0 to T of the constant pressure heat capacity integrated over T. This final term, the C_p term, is calculated within the quasi-harmonic approximation (QHA).

The QHA is essentially the harmonic approximation at different volumes.[?] Due to the computational expense of these calculations, we only performed them for the experimentally observed polymorphs. To simulate different volumes, the crystal structure was fully relaxed at 7 different pressures.[9?] Negative pressures represent thermal expansion and positive pressures represent compression. Relaxations were performed at -0.8, -0.6, -0.4, -0.2, 0.0, 0.2, 0.4 GPa. The harmonic approximation was then performed for each relaxed structure, as described above.

The QHA has been verified for EMs in our previous work,[9] as well as by others.[12–15] Figure S19 shows the constant pressure heat capacity C_p as a function of temperature for the β and α forms of HMX, compared to experimental results from Krien *et al.*,[16] shown in red. Good agreement with experiment is obtained for both polymorphs.

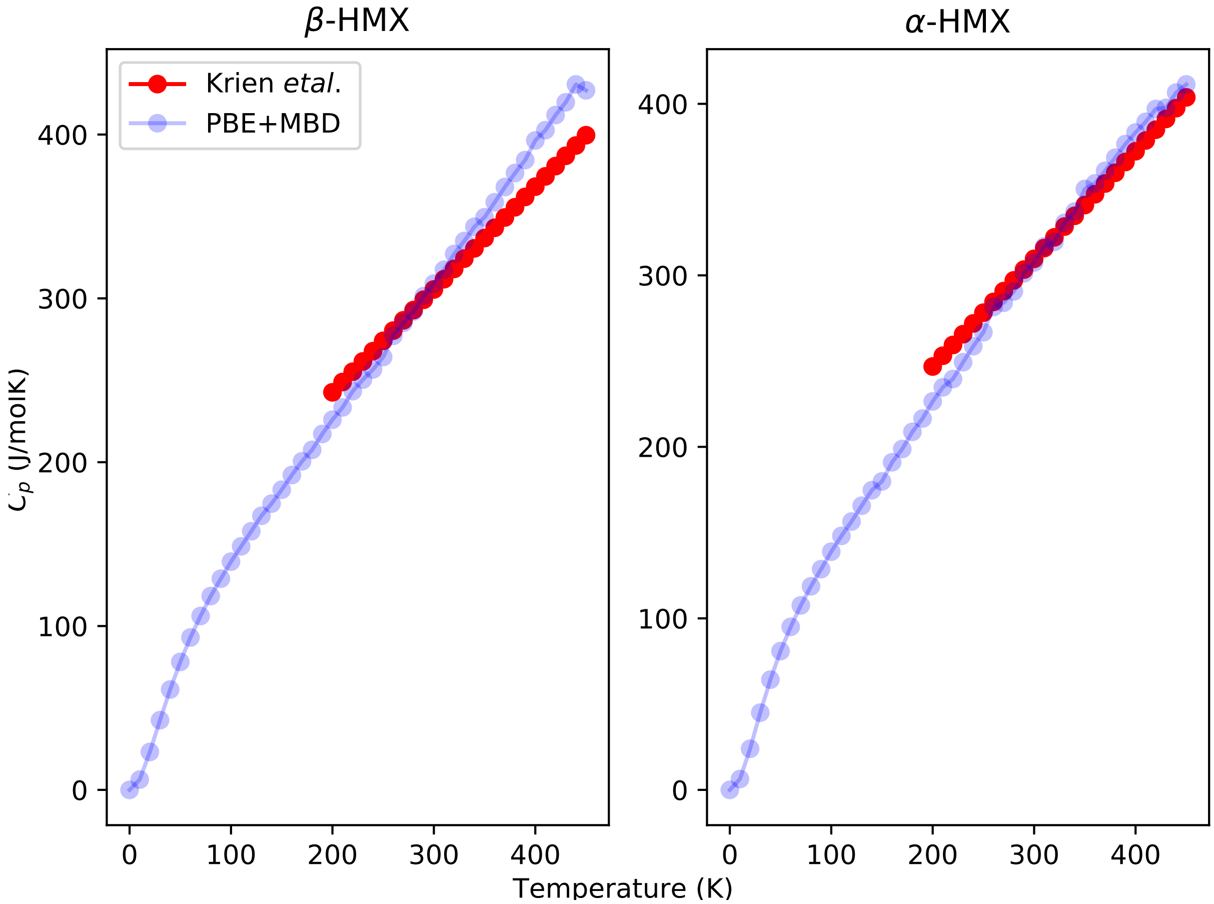

Figure S19: C_p as a function of temperature for the β and α polymorphs of HMX. C_p values were calculated with PBE+MBD within the QHA, shown in blue. Experimental results are shown in red.

The rankings of the two experimental polymorphs with PBE+MBD, PBE0+MBD, PBE0+MBD+ZPE, PBE0+MBD+$E_{vib}(T)$, and PBE0+MBD+ZPE+F_{295} are shown in Figure S20. The α and β forms are shown in red and blue, respectively. The β polymorph is ranked as less stable than the α form at every level of theory, including PBE0+MBD+ZPE+F_{295}, which is the highest level of theory we have ever attempted to calculate for a CSP workflow. However we note that the inclusion of thermal effects explicitly significantly stabilized the β form, supporting our previous work that suggested the assumptions

made in the calculation of the vibrational energy, mainly that molecular crystals undergo little to no thermal expansion as temperature increases, may not be valid for energetic materials.[9]

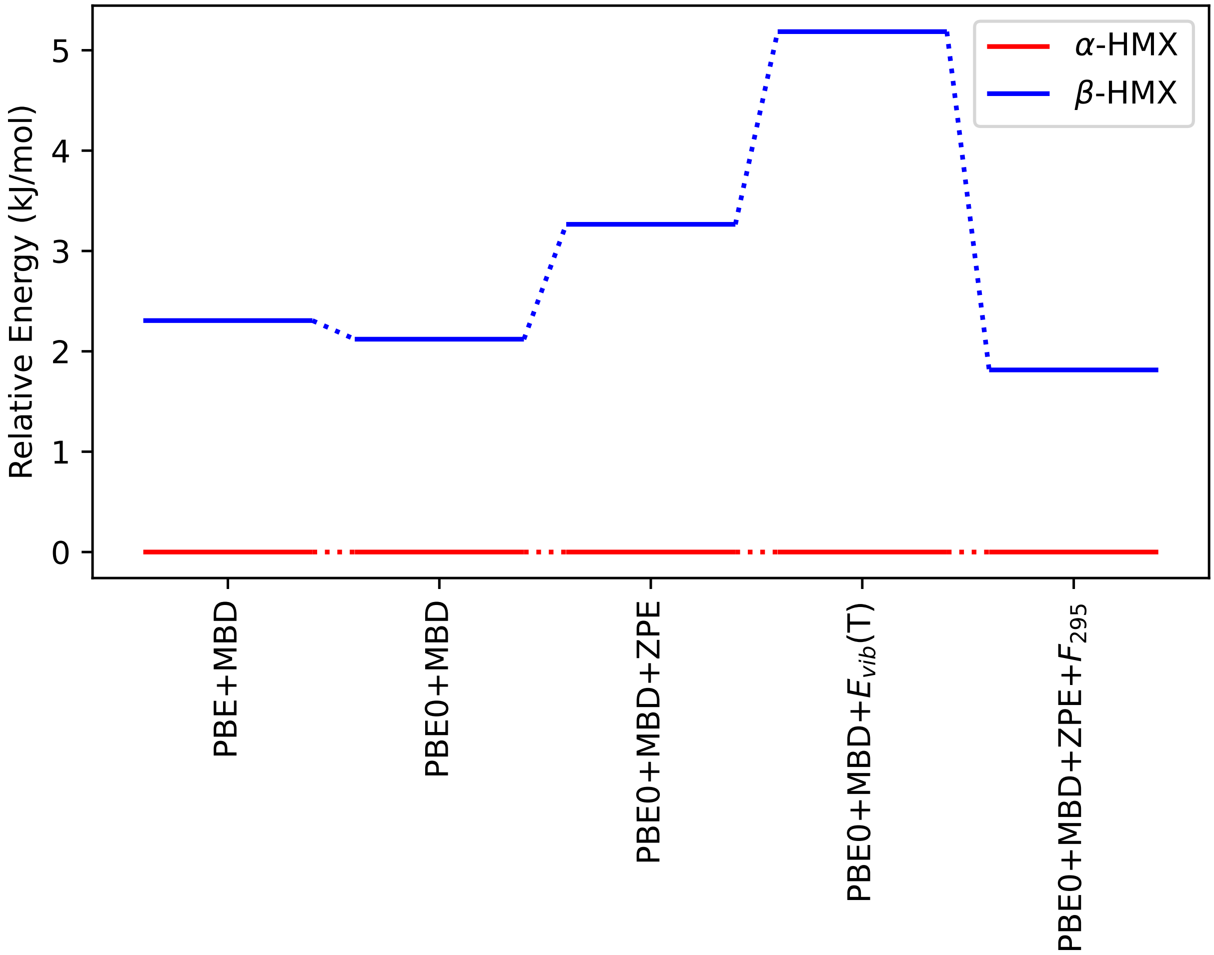

Figure S20: Rankings of the α and β forms of HMX, shown in red and blue, respectively, with increasingly accurate DFT methods. Thermal effects are explicitly considered as calculated within the quasi-harmonic approximation.

8 Computational Cost

As with all computational experiments, CSP requires careful consideration between accuracy and computational cost. For our CSP algorithm, we have chosen dispersion-inclusive DFT due to the high accuracy required for polymorph ranking. With this in mind, we have examined the computational cost of geometry optimizations with PBE+TS while performing GAtor for each material studied here. The results are presented in Table S9 where the average optimization cost, number of optimizations, and the total cost are shown. Calculations were performed on Intel Xeon E5-2695 v4 CPUs with 36 cores per node. As expected, the larger two systems, α-RDX and α-HMX, cost more than the smaller system, LLM-105. While this does not capture the total computational cost, as it excludes PBE+TS optimizations performed in Genarris and the cost of post-processing calculations, this should give a rough estimate of the cost of running GAtor for a semi-rigid small molecule.

Target	Average Optimization Time (s)	Total Optimizations	Total Cost (hours)
LLM-105	8426	959	2245
RDX	15011	1253	5225
α-HMX	17887	880	4372
β-HMX	569	726	115

Table S9: Computational costs for running optimizations with PBE+TS in GAtor.

References

[1] Bier, I.; O'Connor, D.; Hsieh, Y.-T.; Wen, W.; Hiszpanski, A. M.; Han, T. Y.-J.; Marom, N. *CrystEngComm* **2021**, –.

[2] Blum, V.; Gehrke, R.; Hanke, F.; Havu, P.; Havu, V.; Ren, X.; Reuter, K.; Scheffler, M. *Computer Physics Communications* **2009**, *180*, 2175–2196.

[3] Perdew, J. P.; Burke, K.; Ernzerhof, M. *Phys. Rev. Lett.* **1996**, *77*, 3865 – 3868.

[4] Tkatchenko, A.; Scheffler, M. *Phys. Rev. Lett.* **2009**, *102*, 073005.

[5] Tkatchenko, A.; DiStasio, R. A.; Car, R.; Scheffler, M. *Phys. Rev. Lett.* **2012**, *108*, 236402.

[6] Ambrosetti, A.; Reilly, A. M.; DiStasio, R. A.; Tkatchenko, A. *The Journal of Chemical Physics* **2014**, *140*, 18A508.

[7] Adamo, C.; Barone, V. *The Journal of Chemical Physics* **1999**, *110*, 6158–6170.

[8] Yang, S.; Bier, I.; Wen, W.; Zhan, J.; Moayedpour, S.; Marom, N. *The Journal of Chemical Physics* **2020**, *152*, 244122.

[9] O'Connor, D.; Bier, I.; Hsieh, Y.-T.; Marom, N. *Journal of Chemical Theory and Computation* **2022**, *18*, 4456–4471.

[10] Bier, I.; Marom, N. *The Journal of Physical Chemistry A* **2020**, *124*, 10330–10345, PMID: 33253569.

[11] Tom, R.; Rose, T.; Bier, I.; O'Brien, H.; Vazquez-Mayagoitia, A.; Marom, N. *Computer Physics Communications* **2020**, *250*, 107170.

[12] Sorescu, D.; Larentzos, J.; Rice, B.; Brennan, J. *Propellants, Explos. Pyrotech.* **2022**, *47*, e202100338.

[13] Xu, K.; Song, J.; Zhao, F.; Cao, Z.; Ma, H.; Hu, R.; Gao, H.; Huang, J. *Journal of Chemistry* **2007**, *65*, 2827–2831.

[14] Zerilli, F.; Kuklja, M. *AIP Conference Proceedings* **2006**, *845*, 183.

[15] Landerville, A.; Conroy, M.; Budzevich, M.; Lin, Y.; White, C.; Oleynik, I. *Appl. Phys. Lett.* **2010**, *97*, 251908.

[16] Krien, G.; Licht, H.; Zierath, J. *Thermochim. Acta* **1973**, *6*, 465–472.
